# Exploring the Potential of Nanogels: From Drug Carriers to Radiopharmaceutical Agents

**DOI:** 10.1002/adhm.202301404

**Published:** 2023-09-28

**Authors:** Manja Kubeil, Yota Suzuki, Maria Antonietta Casulli, Rozy Kamal, Takeshi Hashimoto, Michael Bachmann, Takashi Hayashita, Holger Stephan

**Affiliations:** ^1^ Helmholtz‐Zentrum Dresden‐Rossendorf Institute of Radiopharmaceutical Cancer Research Bautzner Landstraße 400 01328 Dresden Germany; ^2^ Graduate School of Science and Engineering Saitama University 255 Shimo‐Okubo Sakura‐Ku Saitama 338‐8570 Japan; ^3^ Micro and Nano Systems (MNS) Kasteelpark Arenberg 10, Leuven Heverlee 3001 Belgium; ^4^ Department of Nuclear Medicine Manipal College of Health Professions Manipal Academy of Higher Education Manipal Karnataka 576104 India; ^5^ Faculty of Science & Technology Sophia University 7‐1 Kioi‐cho, Chiyoda‐ku Tokyo 102‐8554 Japan

**Keywords:** cyclodextrin, drug delivery, molecular imaging, nanogels, radiolabeling, theranostics

## Abstract

Nanogels open up access to a wide range of applications and offer among others hopeful approaches for use in the field of biomedicine. This review provides a brief overview of current developments of nanogels in general, particularly in the fields of drug delivery, therapeutic applications, tissue engineering, and sensor systems. Specifically, cyclodextrin (CD)‐based nanogels are important because they have exceptional complexation properties and are highly biocompatible. Nanogels as a whole and CD‐based nanogels in particular can be customized in a wide range of sizes and equipped with a desired surface charge as well as containing additional molecules inside and outside, such as dyes, solubility‐mediating groups or even biological vector molecules for pharmaceutical targeting. Currently, biological investigations are mainly carried out in vitro, but more and more in vivo applications are gaining importance. Modern molecular imaging methods are increasingly being used for the latter. Due to an extremely high sensitivity and the possibility of obtaining quantitative data on pharmacokinetic and pharmacodynamic properties, nuclear methods such as single photon emission computed tomography (SPECT) and positron emission tomography (PET) using radiolabeled compounds are particularly suitable here. The use of radiolabeled nanogels for imaging, but also for therapy, is being discussed.

## Graphical abstract

Nanogels are universally applicable materials mainly being used for drug delivery systems, tissue engineering, and sensor development. More recently, radiolabeled nanogels have also been reported. This class of compounds holds an enormous potential for theranostic applications. In this context, radiolabeled cyclodextrin (CD)‐based nanogels are of particular interest.

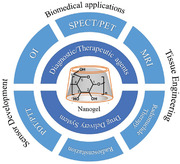

## 1 Introduction

Biomedical research is moving away from standard, one‐size‐fits‐all treatments in favor of more personalized approaches that take into account the conditions and symptoms of individual patients. This development has brought about a new era in medicine, which can be summarized as precision or personalized medicine.^[^ ^1^, ^2^, ^3^ ^]^ The shift is especially evident in oncology, where patients are treated according to the type of tumor as well as to many other parameters like the individual immune system response.^[^ ^4^, ^5^, ^6^, ^7^, ^8^, ^9^ ^]^ The area as a whole has also led to increasing research and development efforts for novel (nano)materials to be used as non‐invasive diagnostic tools and effective therapeutics. Concerning to the latter, current developments are focusing on materials with improved therapeutic efficacy and lower side effects. Despite a number of challenges, such as controlled and reproducible synthesis, effective in vivo targeting and nanotoxicity, faced in the development of nanomaterials, several FDA‐approved nanomedicines are already on the market.^[^ ^10^, ^11^, ^12^, ^13^, ^14^ ^]^ This applies in particular to liquid‐ and polymer‐based nanomaterials, but also to inorganic nanoparticles.

**Figure 1:**
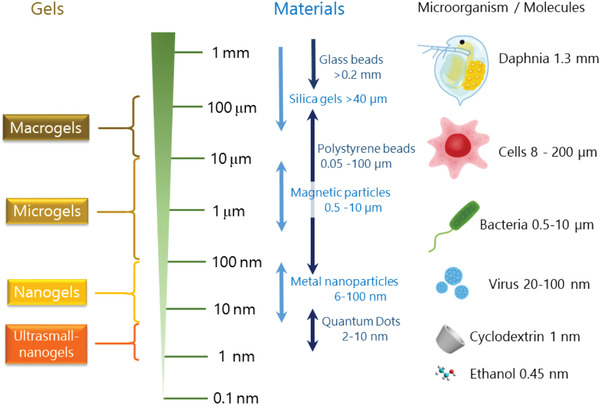
Examples of gels.

A highly interesting class of polymer‐based materials are the so‐called nanogels, because both the size and the properties can be customized in a broad range. Nanogels generally refer to aqueous dispersions of nanosized, physically or chemically cross‐linked polymeric particles.^[^ ^1^ ^]^ The fields of application of nanogels are diverse and include areas such as agriculture, food industry, and the fabrication of organic composite materials. Studies on these interesting nanomaterials, which exploit the functionality of nanogels to achieve improved material properties, represent an active area of research, and many excellent reviews have been published in the last 30 years.^[^ ^6^, ^15^, ^16^, ^17^, ^18^, ^19^, ^20^ ^]^ However, the most extensively studied areas are biomedical applications, spanning the development of customized drug delivery systems, the fabrication of vaccines, tissue engineering, and the field of cancer therapy as a whole. Research on nanogels for medical purposes has currently gained significantly in importance, and a number of review articles have been published in recent years.^[^ ^14^, ^20^, ^21^, ^22^, ^23^, ^24^, ^25^, ^26^, ^27^, ^28^, ^29^, ^30^, ^31^, ^32^ ^]^ Most medically relevant nanogels that have been studied so far have a size range of ten to a few hundred nanometers, which is significantly smaller than biological objects, for example, bacteria or prokaryotic and eukaryotic cells (**Figure** **1**). Derived from that, such nanogels represent ideal transport units in living systems and offer ideal prerequisites for the development of drug delivery systems due to the possibility of encapsulating and releasing drugs and/or diagnostic compounds in a targeted manner.^[^ ^33^ ^]^ In this perspective, the surface of nanogels can be functionalized with a variety of ligands to extend their circulation time in blood and facilitate targeted cell contact.^[^ ^34^, ^35^ ^]^

Besides a multitude of hurdles that have to be overcome for an in vivo application of nanomaterials in general and nanogels in particular, effective in vivo targeting is a major challenge.^[^ ^36^, ^37^, ^38^ ^]^ This requires the engineering of precision nanomaterials and detailed information on biodistribution as well as pharmacokinetic and pharmacodynamic properties.^[^ ^39^ ^]^ Regarding the latter, molecular imaging has proven to be a valuable tool.^[^ ^40^, ^41^, ^42^, ^43^, ^44^ ^]^ With the help of various techniques, including magnetic resonance imaging (MRI), fluorescence optical imaging (OI), ultrasound (US) and nuclear techniques such as single photon emission computed tomography (SPECT) and positron emission tomography (PET), one gains profound insights into the in vivo behavior of nanomaterials. It is worth highlighting that nuclear techniques in particular allow quantitative data on the pharmacokinetic properties of radiolabeled nanomaterials, as well as release protocols of entrapped guest compounds.^[^ ^45^, ^46^, ^47^, ^48^, ^49^, ^50^ ^]^ This has led to intensive studies of in vitro and in vivo properties of nanogels using radiolabels, particularly in recent times.

This review provides a general overview of nanogels and summarizes the main areas of application. Cyclodextrin‐based nanogels are of particular interest, and the main fabrication methods are compiled here, including surface functionalization. The host–guest properties of CD‐based nanogels are described and selected biomedical applications are discussed. The state of research in the field of radioactively labeled nanogels will be presented and the emerging application possibilities in the fields of diagnostics and therapy will be discussed.

## 2 Nanogels

### 2.1 Overview

Polymers comprise a wide range of different natural and synthetic structures and form among others various medically relevant architectures such as nanocapsules, nanospheres, and cross‐linked or self‐organized 3D networks. Depending on the hydrophilic‐lipophilic balance, polymeric structures can be stabilized in different media. Dispersants used in forming polymeric dispersions are broadly classified into water and organic solvents. Nanogels obtained using water as a dispersant are referred to as hydrogels. A hydrogel has a 3D network structure formed by cross‐linked or self‐organized hydrophilic polymers and swells when it contains water. Apart from these structural characteristics, the most important feature of hydrogels is their physical property, i.e., selective permeability. The permeability of hydrogels to molecules smaller than their mesh size is as high as that of water. This property facilitates the movement of small molecules into and out of the gel, as if they were diffusing in water, whereas large molecules (i.e., as large as or larger than the mesh size) are restricted in diffusion by the mesh structure, so their release out of the gel takes a time or they remain in the gel. Hydrogels have unique properties of both liquid and solid, and their swelling and shrinkage behavior, or changes in mesh size, can be controlled by using the volume phase transition of the constituent polymers. The permeability of hydrogels also changes in response to stimuli.

Nanogels have a number of features that predestine them for biomedical applications, such as high hydrophilicity, wide range of functionalization possibilities, and biocompatibility. Moreover, because of their flexible internal structure, nanogels can incorporate various functional molecules and change their sizes in response to external stimuli or under different solvent conditions. Drug delivery systems (DDSs) that utilize this feature have been actively investigated.

Nanogels are composed of hydrophilic functional groups connected by numerous cross‐linkers through several types of interactions: chemical bonding interactions (covalent bonds, coordination bonds), physical interactions (electrostatic interactions, hydrophobic interactions), and supramolecular interactions (combinations of weak interactions). Many review articles have classified nanogels by focusing on their terminal functional groups and cross‐linked structures. The most frequently reported nanogels are those aimed at medical applications. These applications are based on the cell permeability, inclusion ability, and environmental responsiveness of nanogels. Research on nanogels for medical purposes has been extremely active, and a number of review articles^[^ ^23^, ^24^, ^25^, ^27^, ^29^, ^51^, ^52^, ^53^, ^54^, ^55^, ^56^ ^]^ have been published in recent years. In this chapter, we introduce recently published articles (after 2021) categorized according to their intended use, mainly therapeutic applications and tissue engineering (other applications such as foods, agrochemical delivery system, and other unique materials lies beyond the scope of this review).

### 2.2 Biomedical Applications

#### 2.2.1 Drug Delivery

One of the most interesting areas of drug delivery systems (DDS) research is the application of nanogels as carriers of cancer drugs. Doxorubicin (DOX) is the most extensively studied drug in this context. DOX intercalates into DNA of tumor cells, effectively inhibiting or killing the cells. It is poorly soluble in water and almost insoluble in organic solvents. Moreover, DOX needs to be transported directly to the target cancer cells to avoid side effects, and to this end, various DDSs have been proposed.

Lin et al. proposed a DDS that uses nanogels hybridized with gold nanoparticles. Gold nanoparticles were loaded by reducing a hybrid nanogel consisting of a derivative of hyaluronic acid (HA) and cystamine bisacrylamide, with DOX incorporated into the nanogel.^[^ ^57^ ^]^ When the nanogel was placed in a glutathione solution (similar to the environment inside tumor cells), the near‐infrared fluorescence of the gold nanoparticles was enhanced, and the degradation of the nanogel by glutathione caused a simultaneous release of DOX with a decrease in fluorescence.

The development of nanogels containing a mixture of DOX and immunosuppressive agents for combined immunotherapy and chemotherapy has also been reported.^[^ ^58^, ^59^ ^]^ Ma et al. synthesized nanogels encapsulating mannose and DOX, which demonstrated excellent biological compatibility and stability, selective drug release, improved tumor permeability, and prolonged survival due to prolonged blood circulation.^[^ ^59^ ^]^ Tang et al. reported a strategy to fabricate an aptamer‐modified DNA tetrahedron‐based nanogel for combined chemo/gene therapy of multidrug‐resistant tumors.^[^ ^60^ ^]^

The limited permeability of the blood‐brain barrier, which prevents effective delivery of chemotherapeutic agents to the brain, is an important issue. To address this, Song et al. developed a pH/reduction‐sensitive carboxymethyl chitosan nanogel modified by angiopep‐2 (ANG), a targeting peptide, and loaded with DOX.^[^ ^61^ ^]^ This multifunctional nanogel showed high pH sensitivity, ideal stability, and biocompatibility, with a hydrodynamic diameter of about 190 nm, drug loading content of 12.7%, and 24‐hour cumulative release rate of 82.3%, in a simulated tumor microenvironment. Duro‐Castano et al. proposed the use of injectable poly‐amino acid‐based nanogels as a versatile hydrophilic drug delivery platform for the treatment of triple‐negative breast cancer (TNBC)‐associated lung metastasis.^[^ ^62^ ^]^

In addition to DOX, a number of studies have investigated many other drugs that can be loaded into nanogels and delivered to tumors by DDSs. As for the release of drugs, several methods have been reported, including methods based on internal environmental stimuli,^[^ ^63^, ^64^, ^65^ ^]^ temperature stimuli,^[^ ^63^, ^66^ ^]^ external stimuli (ultrasonication),^[^ ^67^, ^68^ ^]^ molecular chaperones,^[^ ^69^ ^]^ pH response,^[^ ^66^, ^70^, ^71^, ^72^ ^]^ and the degradation of nanogels via the dissociation of disulfide bonds.

The oxygen concentration in tumor tissue is often lower than that in normal tissue. By designing nanogels that denature in response to a drop‐in oxygen concentration, some groups tried to achieve drug release at the target tumor. Zhang et al. developed nanogels with a diameter of around 100–160 nm by combining nitroimidazole‐modified hyaluronic acid (HA) with thiolated polyethylene glycol (PEG).^[^ ^65^ ^]^ When these nanogels were placed in a low‐oxygen tumor microenvironment, where nitroimidazole was reduced to aminoimidazole, and the hydrophilic drug was released with the breakdown of disulfide bonds. She et al. prepared azobenzene‐bridged zwitterionic phosphorylcholine nanogels that disintegrate in a low‐oxygen atmosphere; the decomposition of azobenzene into two aniline derivatives allowed for the rapid release of the loaded drug.^[^ ^64^ ^]^ Li et al. developed a DDS based on a composite of a thermosensitive hydrogel and a reactive oxygen species (ROS)‐responsive nanogel.^[^ ^63^ ^]^ By combining the two types of nanogels each encapsulating a drug, multiple active ingredients could be released in the desired order.

Sun et al. introduced ultrasound‐responsive nanogels having a stable structure, which were formed by cross‐linking a copolymer of polylysine and Pluronic F127 by genipin; antibodies capable of binding to the target tissue were grafted onto the nanogels.^[^ ^68^ ^]^ When injected in vivo and treated with ultrasound externally after reaching the target tissue, swelling (329 nm to 516 nm) and softening of the nanogels were induced, leading to deep tumor penetration and drug release.

Mimicking the function of molecular chaperones, Kawasaki et al. created magnetic in vivo protein transport nanogels. In the presence of a magnetic field in vivo, nanogels are assembled because of the encapsulated iron oxide nanoparticles.^[^ ^69^ ^]^ The nanogels also contained saporin, an anticancer protein, which was rapidly released by the exchange reaction with serum protein. The evaluation using an oral cancer model revealed a reduction in tumor volume and suppression of tumor re‐growth, with no change in body weight.

#### 2.2.2 Photothermal and Photodynamic Therapy

Photothermal therapy (PTT) and photodynamic therapy (PDT) have been the subject of much research in recent years. Photo‐responsive molecules or particles are adsorbed on the targeted cells (e.g., cancer cells), and light energy (e.g., near‐infrared light) is applied from outside the body to release or generate (by chemical reaction) active ingredients, such as drugs. Research on the application of nanogels for selective tissue adsorption, confinement of photo‐responsive molecules, and drug release response is under way.

Qin et al. developed a hydrogel for combined glutathione‐activated chemotherapy and PDT for the control of ROS in cancer cells.^[^ ^73^ ^]^ Lactate oxidase (LOx) and catalase (CAT) integrated into Fe_3_O_4_ nanoparticle/indocyanine green (ICG) co‐loaded hybrid nanogels were designed for ROS‐induced tumor therapy. The hybrid nanogels with an optimized ratio of LOx to CAT fatally damaged tumor cells by significantly increasing ROS levels in the cells, thus markedly inhibiting tumor growth. Cai et al. presented an efficient and safe PTT/PDT multi‐mode synergistic antimicrobial strategy for treating biofilm infections.^[^ ^74^ ^]^ The nanoplatform was constructed by physically encapsulating photoresponsive ICG and [MnBr(CO)_5_] into bipyridine derivative‐modified peptide dendrimer‐based nanogels approximately 200 nm to 250 nm in size. Large amounts of CO could be generated by the synergistic effects of PTT and PDT. The generated CO promoted biofilm penetration of ICG and enhanced the antibacterial and biofilm eradication activity of PTT and PDT. Moreover, the inflammatory response caused by bacterial infection was also significantly reduced.

Wu et al. proposed a system based on tetrafluorophenyl bacteriochlorin (FBC) for PDT. FBC is a highly stable bacteriochlorin analog obtained by a one‐step reduction of tetrafluorophenyl porphyrin (TFPP).^[^ ^75^ ^]^ Biocompatible FBC nanogels (80–200 nm) were formed directly by mixing FBC with thiolated PEG. The FBC nanogels were activated at a biopermeable near‐infrared light wavelength of 750 nm, resulting in not only oxygen concentration‐dependent ^1^O_2_ generation but also less oxygen‐dependent O_2_^−^ and •OH generation, achieving excellent PDT in hypoxic tumors.

Zhao et al. proposed a nanogel designed for achieving PTT and PDT efficacy by combining carbon dots and poly(*N*‐isopropylacrylamide).^[^ ^76^ ^]^ Large composite particles were designed to accumulate at the tumor site, where partial degradation occurred upon irradiation with 660 nm light. Both heat and ROS were generated by the carbon dots, attacking the tumor cells. Furthermore, under low pH and high‐temperature conditions (i.e., inside the tumor), the disulfide bonds in the cross‐links were cleaved by glutathione (GSH), thereby facilitating the release of the active ingredient and the ejection of the nanogel. Ma et al. prepared a nanogel with a similar mechanism^[^ ^77^ ^]^ using the reductive cross‐linking of purpurin 18 (P18) and 10‐hydroxy camptothecin (HCPT); this nanogel had a relatively small size (67 nm). The high GHS concentration in the tumor microenvironment caused the nanogel's disulfide bonds to break, releasing HCPT and P18. Laser irradiation of the released P18 at 660 nm stimulated the generation of ROS. P18 also acted as a near‐infrared fluorescence probe and a magnetic resonance (MR) imaging agent.

Curcumin has low hydrophilicity and susceptibility to photodegradation, and is often used as a model compound for drug delivery, as well as a guest molecule in the development of host compounds. Dinari et al. developed nanogels measuring approximately 100 nm by graft copolymerization of temperature‐responsive *N*‐isopropylacrylamide (NIPAM) and *N*,*N*‐dimethylaminoethyl methacrylate (DMAEMA) on a lignin substrate, and evaluated their adsorption capacity and release behavior with curcumin as a model drug.^[^ ^78^ ^]^ Luckanagul et al. prepared spherical nanogels (diameter 150 nm), which is HA‐grafted (*N*‐isopropylacrylamide), and evaluated the water solubility of curcumin and its uptake by NIH‐3T3 cells.^[^ ^79^ ^]^ Guo et al. produced micelles of curcumin with a cholesterol‐modified natural polysaccharide (angelica polysaccharide: APS), which was disulfide‐linked and encapsulated in the erythrocyte membrane (approximately 170 nm in size).^[^ ^80^ ^]^ The capsules prepared with APS showed higher tumor‐tissue‐search capacity and higher anti‐hepatitis efficiency than those prepared with HA.

Zhao et al. developed a nanogel as a mucolytic agent for the treatment of asthma.^[^ ^81^ ^]^ This nanogel, which consists of arginine‐grafted chitosan and tris(2‐carboxyethyl)phosphine (TCEP), can be administered via a nebulizer. The nanogel prevented the aggregation of mucin, i.e., the cause of mucus clogging in the nasal cavity and elsewhere, via two types of actions, i.e., the electrostatic interaction at the chitosan moiety and disulfide bond cleavage by TCEP, which effectively inhibited the growth of two bacterial species.

Intranasal vaccines for *Streptococcus pneumoniae* have also been developed. Yuki et al. synthesized cationic cholesteryl group‐bearing pullulan (CHP), a polysaccharide with cationic cholesterol side chains, and obtained small pullulan nanogels with a diameter of 26 nm to 30 nm via self‐assembly.^[^ ^82^ ^]^ The circular dichroism spectra confirmed that these nanogels trapped the pneumococcal vaccine, while retaining its chirality without denaturation. Nakahashi‐Ouchida et al. verified the efficacy of this nanogel‐based intranasal vaccine in macaque monkey experiments.^[^ ^83^ ^]^

A nanogel that inhibits biofilm formation on the nasopharynx by nontypeable *Haemophilus influenzae* (NTHi) has also been developed, utilizing the same pullulan backbone as that described above,^[^ ^84^ ^]^ i.e., a CHP conjugated to the NTHi surface antigen (P6). Mouse experiments showed that the nasal administration of the nanogel induced P6‐specific IgA and inhibited NTHi biofilm formation.

#### 2.2.3 Tissue Engineering

Nakai et al. used freeze‐dried nanogel‐cross‐linked porous (FD‐NanoCliP) polysaccharide sheets/fibers as a scaffold material to evaluate the conversion ability of human dermal fibroblasts (HDFs) into osteoblasts.^[^ ^85^ ^]^ FD‐NanoCliP sheets were prepared by cross‐linking cholesterol partially bearing pullulan with acroyl groups and PEG having terminal thiol groups to form a NanoCliP gel. This gel was then converted into a FD‐NanoCliP sheet by freeze‐thawing, freeze‐drying, and soaking in a solution. As cells can be prepared in sheet or fiber form, cellular components can be incorporated not only into the nanogel but also into the surface shape of the porous part. The performance of the FD‐NanoCliP gel as a scaffold was compared with a conventional atelocollagen scaffold. A highly calcified substance formation capacity suggested the potential of using the gel as a material for the rapid repair of large bone defects. The behavior during bone formation was also studied using Raman and ATR‐FTIR spectroscopy by Adachi et al. It was confirmed that the regenerated tissue contained more amounts of cartilage matrix tissue.^[^ ^86^ ^]^

Cyclodextrin (CD)‐based nanogels are a special class with exceptional properties that have gained in importance in recent years. Cyclodextrins are known as characteristic sugar molecules, possessing hydrophilic exterior and hydrophobic interior cavities, which enable them to incorporate various guest molecules and could be a motif for providing additional functions to nanogels. Such CD‐based nanogels are described in detail in the following chapter.

## 3 CD‐Based Nanogels

### 3.1 Synthesis Methods

**Figure 2:**
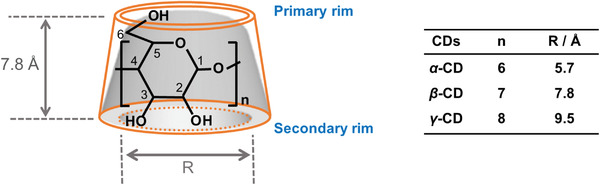
Cartoon illustration of CD structure.

This section summarizes different synthetic strategies for CD‐based nanogels and their general properties, with a focus on size. CDs are water‐soluble cyclic oligosaccharides with conical cylinder structures, and their nano‐order hydrophobic cavities can encapsulate a variety of hydrophobic compounds.^[^ ^87^, ^88^ ^]^ CDs promote the solubilization of hydrophobic compounds in an aqueous solution by encapsulation; this is a beneficial property from the perspective of pharmaceutical applications, given that 40% of currently marketed drugs and approximately 90% of new drug candidates are insoluble at their therapeutic concentrations in physiological fluids.^[^ ^89^ ^]^ Owing to their unique properties, CDs have been used in various research areas including solubilizers,^[^ ^90^ ^]^ drug delivery,^[^ ^91^ ^]^ stabilizers for therapeutic proteins,^[^ ^92^ ^]^ separation,^[^ ^93^ ^]^ molecular machines,^[^ ^94^ ^]^ reaction media,^[^ ^95^, ^96^ ^]^ molecular recognition,^[^ ^97^ ^]^ chemosensors,^[^ ^98^, ^99^, ^100^, ^101^, ^102^, ^103^, ^104^, ^105^ ^]^ and food ingredients.^[^ ^106^ ^]^ The most common CDs, α‐CD, β‐CD, and γ‐CD, are composed of six, seven, and eight D‐glucopyranose units, respectively. The sizes of the CD cavities vary depending on the number of D‐glucopyranose units contained in the CD, as summarized in **Figure** **2**.^[^ ^88^ ^]^ CDs contain two types of hydroxyl groups, primary and secondary hydroxyl groups, located on the smaller and larger rims of CDs, respectively. The primary hydroxyl groups (C6‐OH) can rotate, resulting in the decreased effective diameter of the primary rim compared with that of the secondary rim. These hydroxyl groups are the most basic of all hydroxyl groups in the D‐glucopyranose unit, and various functional groups can be incorporated through nucleophilic reactions. In contrast, the secondary hydroxyl groups (C2‐OH and C3‐OH) are engaged in rigid hydrogen bonding and can also form reactive oxyanions. As such, the primary and secondary faces of CDs can be modified with various functional groups by simple organic reactions.^[^ ^87^ ^]^ Moreover, CDs modified with reactive functional groups can be used to prepare polymeric materials with appropriate linkers.^[^ ^33^ ^]^

**Figure 3:**
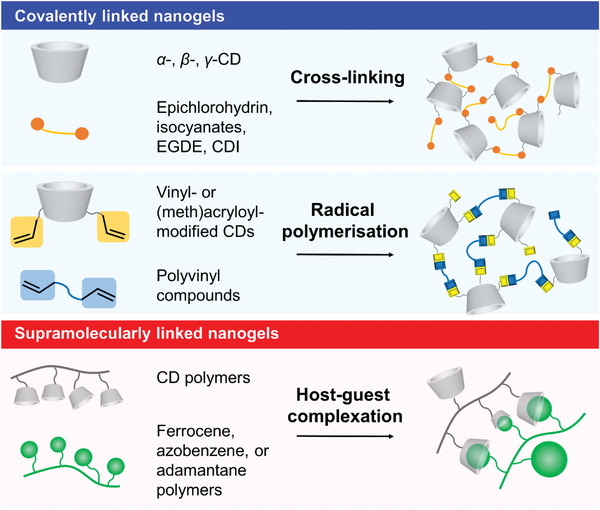
Cartoon illustrations of CD nanogels and their preparation methods.

The advantages of CD‐based nanogels include biocompatibility, low toxicity, and hydrophobic compound‐loading capability.^[^ ^107^, ^108^, ^109^ ^]^ Various CD‐based nanogels have been developed to exploit these attractive features and are used for biomedical applications such as drug delivery‐based therapies,^[^ ^109^, ^110^ ^]^ diagnostics,^[^ ^107^ ^]^ and bio‐imaging.^[^ ^111^, ^112^ ^]^ A wide range of preparation methods have been developed to impart various types of characteristics to CD‐based nanogels, which are extensively summarized by several comprehensive and specialized reviews.^[^ ^109^, ^113^ ^]^ There are two main design strategies for the preparation of CD‐based nanogels: 1) covalent linkage of CDs, and 2) supramolecular linkage of CDs (**Figure** **3**). Covalently linked CD nanogels are composed of 3D networks formed by the polymerization reactions of CDs (or modified CDs) with organic linkers (e.g., cross‐linking, radical polymerization). Because CDs in nanogels are linked through covalent bonds, this strategy provides highly stable CD nanogels and allows for the control of nanogel size by adjusting the reaction conditions. In contrast, supramolecularly linked CD nanogels are formed via noncovalent assembly (e.g., host–guest complexation). This strategy offers a simple and facile preparation method for nanogels. Compared with covalently linked CD nanogels, supramolecularly linked CD nanogels are fragile, being extremely sensitive to external stimuli such as temperature, pH, and added molecules. Therefore, stimulus‐responsive nanomaterials can be easily architected for the design of drug‐releasing systems.^[^ ^114^ ^]^

#### 3.1.1 Cross‐Linking

**Figure 4:**
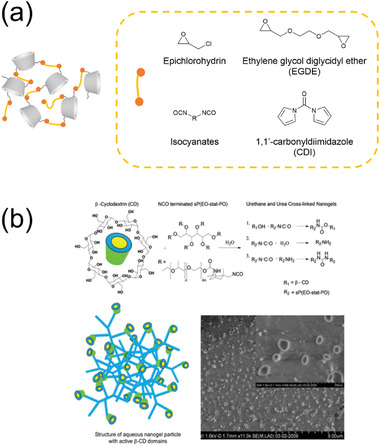
a) Illustration of host–guest complexation‐based nanogels and structures of typical cross‐linkers. b) Illustration of the synthesis of nanogels cross‐linked by six‐arm isocyanate‐terminated linkers, together with Cryo‐FESEM images of swollen nanogels. Reproduced with permission.^[^ ^116^ ^]^ Copyright 2012, American Chemical Society.

Native CD monomers have many reactive hydroxyl groups that can be linked with multiple CD units using cross‐linkers (**Figure** **4**a), e.g., epichlorohydrin,^[^ ^33^, ^115^ ^]^ isocyanates,^[^ ^111^, ^116^ ^]^ ethylene glycol diglycidyl ether (EGDE),^[^ ^117^ ^]^ and 1,1′‐carbonyldiimidazole (CDI).^[^ ^118^, ^119^ ^]^ As regards cross‐linked CD nanogels, the CD monomers are often linked via organic reactions using general procedures, such as heating or stirring reactants at room temperature. In 1997, Renard et al. reported the preparation of water‐soluble β‐CD polymers using epichlorohydrin as a cross‐linker to form ether bonds. The reaction conditions, which involved stirring β‐CD and epichlorohydrin at 30 °C under basic conditions, were fully investigated and established for obtaining nanogels with the desired properties.^[^ ^115^ ^]^ Following this report, Anand et al. developed β‐CD‐based nanogels with a diameter of 15 nm.^[^ ^120^ ^]^ They evaluated these nanogels as drug delivery carriers for DOX and artemisinin. Polyisocyanate compounds can also be used as cross‐linkers for CD nanogels, as the isocyanate groups react with the hydroxyl groups to form polyurethane polymers. Kettel et al. reported the preparation of β‐CD nanogels composed of six‐arm isocyanate‐terminated linkers by stirring reactants at room temperature in water under tenside‐free conditions (Figure 4b).^[^ ^116^ ^]^ This preparation method provided uniform nanogels measuring 100 to 300 nm in diameter with narrow size distributions. They also found that larger nanogels could be obtained by increasing the β‐CD content in the reaction mixture. Cross‐linked CD nanogels can be easily functionalized by incorporating appropriate functional groups. Deng et al. reported fluorescent β‐CD nanogels having two fluorophore units, 4‐amino‐7‐nitro‐1,2,3‐benzoxadiazole and spiropyran, cross‐linked by 1,6‐hexamethylene diisocyanate.^[^ ^111^ ^]^ These nanogels were prepared by heating the reaction mixture at 70 °C for 6 h, followed by stirring in the presence of sodium dodecyl sulfate overnight. The obtained dispersion was purified by dialysis. These nanogels were color‐switchable, i.e., their fluorescence could be reversibly regulated by ultraviolet (UV) or visible light irradiation.

**Figure 5:**
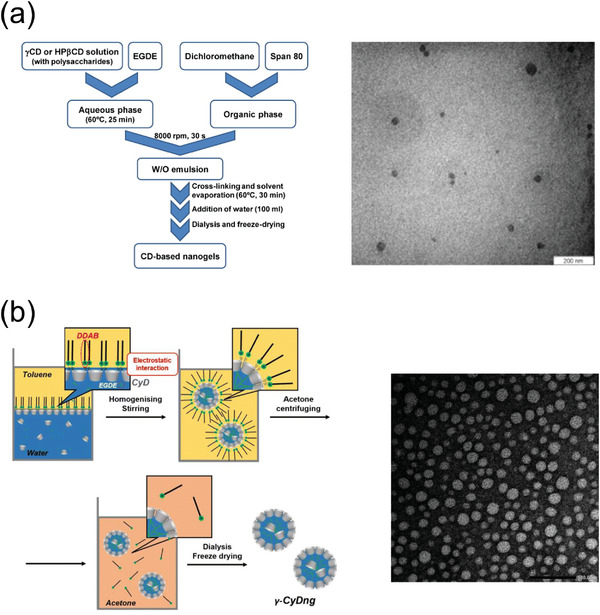
a) Schematic illustration of the synthesis and TEM image of CD‐based EGDE nanogel. Reproduced with permission.^[^ ^117^ ^]^ Copyright 2012, Elsevier. b) Schematic illustration of the synthesis and TEM image of ultrasmall *γ*‐CD nanogel. Reproduced with permission.^[^ ^122^ ^]^ Copyright 2023, the Royal Society of Chemistry.

Typical organic reactions sometimes require harsh conditions, resulting in a nonuniform size distribution and the structural deformation of nanogels. Some researchers have developed a preparation method for CD‐based nanogels using inverse emulsion polymerization. In this method, water‐in‐oil emulsions are formed in two‐phase solvent systems by homogenization or vigorous stirring under gentle conditions.^[^ ^113^, ^121^ ^]^ An oil‐soluble surfactant is usually added to stabilize the surface of inverse emulsion particles in a continuous oil phase. In aqueous droplets, CD monomers are cross‐linked to provide size‐controlled nanogels. The oil phase and the surfactant can be removed by dialysis or centrifugation, and the nanogels can then be obtained in powder form by freeze‐drying. Moya‐Ortega et al. prepared hydroxypropyl β‐CD and γ‐CD nanogels by inverse emulsion polymerization using Span 80, a surfactant, in a two‐phase solvent of sodium hydroxide aqueous solution and dichloromethane (**Figure** **5**).^[^ ^117^ ^]^ The dichloromethane phase contained Span 80, and the aqueous phase contained CDs and the cross‐linker EGDE. The two‐phase solution was homogenized, and subsequently, the formed emulsions were stirred at 60 °C for 30 min. CD‐based nanogels with various diameters (8–800 nm) were obtained by varying the surfactant concentration. Recently, Takeuchi et al. prepared ultrasmall *γ*‐CD‐based nanogels by an inverse emulsion method using a cationic surfactant, [dilauryl(dimethyl)]ammonium bromide (DDAB).^[^ ^122^ ^]^ In a binary solvent system of toluene and sodium hydroxide solution, *γ*‐CD and EGDE were homogenized with DDAB and 1‐hexanol for 15 min at room temperature (Figure 5). The homogenized suspension was stirred at room temperature for 27 h, then, the formed nanogels were purified by dialysis and membrane filtration. Uniformed nanogels with sizes of 10 nm were confirmed by dynamic light scattering (DLS) and transmission electron microscopy (TEM), showing 1) high dispersity in water, 2) strong hydrophobic environment inside the nanogels, and 3) superior inclusion affinities for a hydrophobic compound compared with native γ‐CD.

#### 3.1.2 Radical Polymerization

Another method for preparing CD polymer networks is radical polymerization, in which vinyl‐ or (meth)acryloyl‐modified CDs and other vinyl monomers are copolymerized. Radical polymerization offers a means to effectively link vinyl compounds, furnishing CD polymers with excellent yields.^[^ ^123^ ^]^ Kettel et al. synthesized reactive CDs (α‐, β‐, and γ‐CDs) bearing vinyl groups and polymerized them with N‐vinylcaprolactam.^[^ ^124^ ^]^ The reactants were mixed in water in the presence of an initiator, and the mixture was heated at 70 °C for 12 h without a surfactant. The resulting mixture was purified by ultrafiltration, affording nanogels of 124 nm to 454 nm in diameter. Full screening of preparation conditions revealed that 1) reactions with higher CD contents provide smaller nanogels, and 2) reactions with CDs having a larger number of vinyl groups provide smaller nanogels (down to 45 nm in diameter). The same group also prepared swelling α‐, β‐, and γ‐CD nanogels using poly(methyl methacrylate) as a linker under various reaction conditions.^[^ ^111^ ^]^ In this preparation, CD methacrylates were heated with methyl methacrylate at 65 °C for 8 h in methanol, with azobisisobutyronitrile as an initiator. The nanogels were purified by dialysis. They also found that the higher the CD content and the larger the number of vinyl groups per CD molecule, the smaller the nanogels (72–726 nm in diameter). Moreover, the sizes of these nanogels increased in organic solvents. Chen et al. reported single‐chain polymeric CD nanogels prepared by reversible addition–fragmentation chain transfer (RAFT) polymerization using a chain transfer agent, N,N‐dimethyl acrylamide (block polymers), and an inclusion complex of vinyl‐β‐CD and vinyl‐adamantane (cross‐linkers).^[^ ^125^ ^]^ This preparation method provided ultrasmall nanogels with a hydrodynamic diameter of 7 nm. Interestingly, the nanogels could be unfolded into a single‐chain conformation by the addition of a small amount of competitive adamantane, thereby increasing their diameter to 8.1 nm. Various applications of this nanogel system have been demonstrated, including drug delivery and biomaterials.

#### 3.1.3 Host–Guest Complexation

**Figure 6:**
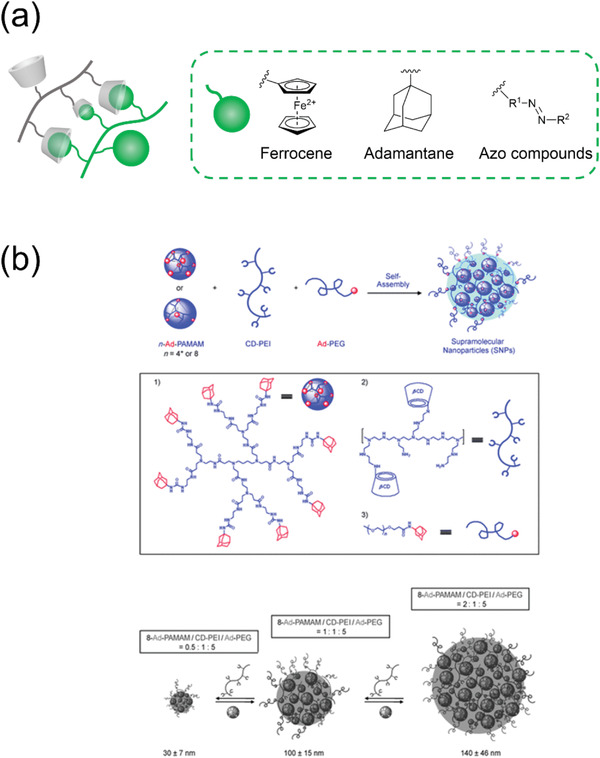
a) Illustration of host–guest complexation‐based nanogels and structures of typical guest units. b) Illustration of the synthesis of CD‐adamantane nanogels.^[^ ^131^ ^]^ Reproduced with permission.^[^ ^131^ ^]^ Copyright 2009, Wiley‐VCH GmbH.

This approach, so‐called “key‐lock assembly,”^[^ ^109^, ^113^ ^]^ exploits the unique property of CDs to form inclusion complexes with hydrophobic compounds. The host–guest complexed CD nanogels can be formed in a spontaneous manner via noncovalent interactions, achieving the desired size and charge. The host–guest complexation system is a promising candidate for the design of DDSs to encapsulate and transport various biologically active compounds, given the biocompatibility and ease in preparation of these materials.^[^ ^126^, ^127^ ^]^ In addition, because of their fragile structure, stimulus‐responsive nanomaterials can be architected on the basis of the host–guest complexation of CD nanogels.^[^ ^128^ ^]^ The key combinations for the nanogel formation are CD‐hydrophobic alkyl chains,^[^ ^129^ ^]^ CD‐adamantane,^[^ ^130^, ^131^ ^]^ CD‐ferrocene,^[^ ^132^ ^]^ and CD‐azo compounds (**Figure** **6**a).^[^ ^132^ ^]^ Gref et al. were the first to establish an easy preparation method to obtain CD nanogels of this type; they used a β‐CD epichlorohydrin polymer and hydrophobic alkyl chain‐grafted dextran to prepare stable nanogels with diameters of approximately 200 nm, with an excellent yield of 95%.^[^ ^129^ ^]^ They also evaluated the drug loading capacity of these nanogels. The same group further investigated the spontaneous formation of the nanogels^[^ ^133^ ^]^ and found that 1) the nanogel formation was entropy‐driven and 2) the nanogel diameter (60–160 nm) was independent of the degree of lauroyl substitution on dextran. Wang et al. established a preparation method for size‐controlled nanogels composed of β‐CD‐grafted branched polyethylenimine and adamantane‐modified first‐generation polyamidoamine dendrimer (Figure 6b).^[^ ^131^ ^]^ In this preparation, adamantane‐functionalized PEG was added to 1) suppress nanogel growth and 2) impart water solubility. By controlling the ratio of the three reagents, CD nanogels with diameters ranging from 30 to 450 nm could be obtained. Using a similar method, Chali et al. prepared host–guest complexed CD nanogels composed of the following three components: adamantane‐conjugated poly‐L‐lysine (guest polymer), CD‐grafted linear poly‐L‐lysine (host polymer), and adamantane‐terminated poly‐L‐lysine (stopper).^[^ ^130^ ^]^ Mixing these components together in water for 30 min, the desired nanogels were easily obtained. Moreover, the size of the nanogels could be controlled by tuning the length of the guest linker (180–355 nm). Furthermore, it was found that 1) longer guest polymers produced larger nanogels, and 2) nanogels obtained from star‐shaped guest polymers were smaller than nanogels prepared using linear guest polymers. Notably, these nanogels could be loaded with a fluorescent compound or DNA. As proteases trigger nanogel degradation, enzyme‐responsive nanomaterials could be designed using this nanogel system.

### 3.2 Advances in Biomedical Applications

Nanogels’ intrinsic modular design makes them ideal for biomedical applications, allowing quick component exchange without system revamping.^[^ ^134^, ^135^ ^]^ CD‐based nanogels, with supramolecular host–guest interactions, offer versatility in various applications, mimicking biological species like enzymes and nucleic acids.^[^ ^136^ ^]^ Their dynamicity, reversibility, and biocompatibility make them promising for biomedical use.^[^ ^137^, ^138^ ^]^ CD's FDA approval for personalized care, drug formulations, and food industry further enhances their appeal.^[^ ^139^ ^]^

This paragraph highlights CD‐based nanogels in drug delivery, while upcoming sections will explore their applications in tissue engineering and sensing.

#### 3.2.1 Drug Loading and Drug Release

**Scheme 1:**
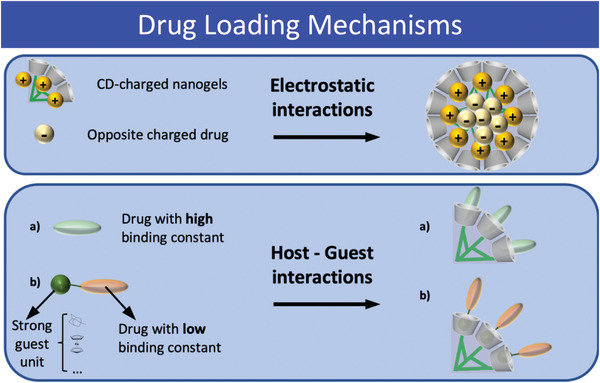
Different drug loading mechanisms in CD‐based nanogels, electrostatic interactions (top); host–guest interactions (bottom).

CD‐based nanogels offer a solution to the limited solubility of drugs in physiological fluids.^[^ ^89^ ^]^ The hydrophilic nature of the polymeric chains in nanogels provides an appropriate environment for drugs to reach the target release site effectively. However, the drawbacks of limited drug loading efficiency and suboptimal control of drug release must be addressed. Supramolecular chemistry of CDs comes to the rescue by integrating hydrophilic moieties into the nanogel's polymeric network, forming inclusion complexes for more efficient drug loading and controlled release, preventing the “burst effect.” Drugs are loaded into CD‐based nanogels through electrostatic and host–guest interactions, where the latter involves direct inclusion in the CD cavity or chemical modification with a strong guest like adamantane (**Scheme** **1**).^[^ ^136^, ^140^, ^141^, ^142^ ^]^ This approach holds promise for enhancing drug delivery efficacy and overcoming solubility challenges in pharmaceutical applications.

**Scheme 2:**
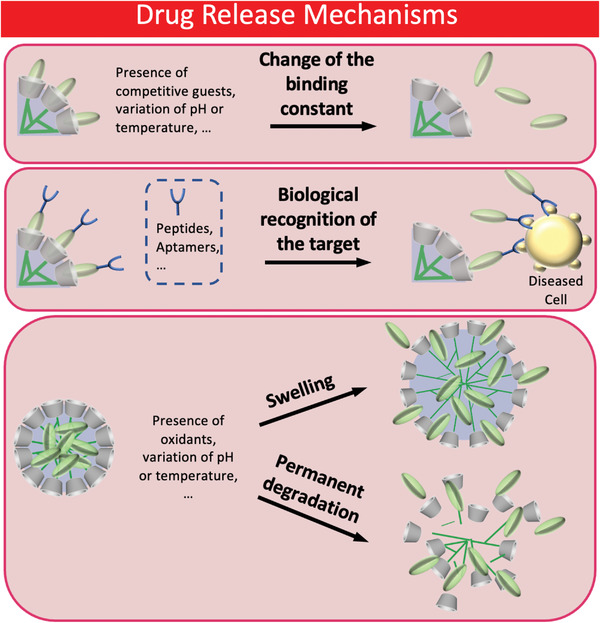
Different drug release mechanisms: change of the binding constant (top); functionalization of nanogels with biological entities for the recognition of diseased cells (middle); temporal swelling or permanent degradation of nanogels (bottom).

Instead, the drug‐release mechanisms of CD‐based nanogels can be regulated by changing the binding constant, by functionalizing the nanogels with specific biological entities that recognize the target, or through temporal loosening/permanent degradation of the polymeric network (**Scheme** **2**).

CD‐based nanogels can effectively encapsulate charged drugs through electrostatic interactions, commonly used in gene delivery for DNA and RNA fragments with negative charges due to phosphate groups. For instance, Davis developed CNP nanogels (50–70 nm) containing a short polycation that interacted electrostatically with synthetic microRNA sequences, enabling correct siRNA encapsulation for anti‐cancer therapy.^[^ ^143^ ^]^ These nanogels selectively accumulated in tumor tissues via the EPR effect, releasing siRNA to interfere with specific mRNA genes.^[^ ^144^ ^]^ Similar polyplex systems using CD‐modified cationic moieties have been designed for gene and drug delivery, such as Ang et al.'s nanogels assembled from CD‐modified PAA and DOX, forming electrostatic bonds between DOX's positive amino groups and PAA's negative carboxyl groups. ^[^ ^145^ ^]^

To encapsulate hydrophobic drugs within CD‐based nanogels, two approaches are utilized. In the first, a supramolecular network is formed based on CDs, as demonstrated by Davis^[^ ^146^ ^]^ They designed self‐assembled nanogels by co‐grafting short PEG polymer chains with β‐CDs and camptothecin (CPT), an essential DNA replication inhibitor. The CPT's inclusion in CD cavities allowed the nanogels to form via reciprocal interactions between the polymer chains, resulting in nanogels sized 30–40 nm. This approach preserved the drug's active form and improved nanogel solubility through PEG's stealth properties. Furthermore, the small size of nanogels minimized immunogenicity, prolonging their circulation time in blood. The nanogels released CPT in tumor cells via the EPR effect, inhibiting replication and leading to nanogel degradation and excretion through the kidneys. Gref et al. developed nanogels by mixing an aqueous solution of β‐CD polymer with a hydrophobically modified dextran grafted with alkyl moieties. The nanogels’ formation involved half of the CDs in the inclusion of dextran's alkyl chains, while the other half was kept free for the inclusion of benzophenone (BZ), a hydrophobic drug. The inclusion of BZ and hydrophobic‐hydrophobic interactions stabilized the complex and improved drug loading efficiency.^[^ ^147^ ^]^ Additionally, Zhang et al. adopted a different approach by functionalizing drugs with high‐affinity guest molecules. They used CaCO_3_ as a template to self‐assemble CD‐dextran with dextran grafted with an adamantane moiety, encapsulating dextran within the nanogels through host–guest interactions with CDs’ free cavities.^[^ ^148^ ^]^

Effective drug delivery is realized when drug molecules are released to the target tissue in a consistent manner after circulating in the bloodstream under stable conditions. In this regard, the size of nanogels is a critical parameter for evaluating the effectiveness of drug delivery. Indeed, nanogels less than 200 nm in size are guaranteed to have a long circulation time in blood.^[^ ^149^, ^150^ ^]^ There are three mechanisms of drug release, which are based on the following characteristics: change of the binding constant of the supramolecular complex, functionalization of nanogels with specific biological entities, and loosening or permanent degradation of the polymeric network.

Drug release in CD‐based nanogel systems often relies on changes in the binding constant, achieved through various triggers such as binding competition or environmental conditions. For example, Ma et al. designed self‐assembled β‐CD nanogels triggered by the inclusion of tyrosine into the CD cavity via host–guest complex formation.^[^ ^151^ ^]^ In the presence of specific moieties, tyrosine was displaced from the CD cavities, weakening the inter‐tyrosine hydrogen bonding and causing disassembly (Scheme 1). Other triggers involve varying pH or temperature.

Tseng et al. developed size‐controlled gold nanoparticles (Au‐NPs) using CD‐grafted branched polyethylenimine (CD‐PEI) and adamantane‐grafted 2 nm Au colloids (Ad‐AuNPs). By adjusting the CD‐PEI to Ad‐AuNPs ratio, stable nanogels of different sizes were obtained. However, at higher temperatures, the binding constant between Ad and the CD cavity decreased, leading to nanogel disassembly. These findings suggested potential application in photodynamic therapy.^[^ ^152^ ^]^

The third mechanism of drug release involves the temporary loosening or permanent degradation of the polymeric network. Jung et al.^[^ ^153^ ^]^ synthesized nanogels made of vinyl ferrocene and methacrylate β‐CDs that swell/de‐swell reversibly based on the presence of chemical oxidants like H_2_O_2_, triggering drug release, especially in tumor tissues.^[^ ^153^ ^]^ Zhang et al. achieved DOX release through polymer swelling due to PDMAEMA protonation under acidic conditions, showing a triphasic trend of drug release.^[^ ^154^ ^]^ Degirmenci et al. reported nanogels with disulfide‐containing bis‐adamantane cross‐linkers, degrading permanently upon exposure to GSH, an endogenous reducing agent found in tumor cells, which doubled DOX release at acidic and neutral pH.^[^ ^155^, ^156^ ^]^

#### 3.2.2 Tissue Engineering Using CD‐Based Nanogels

**Figure 7:**
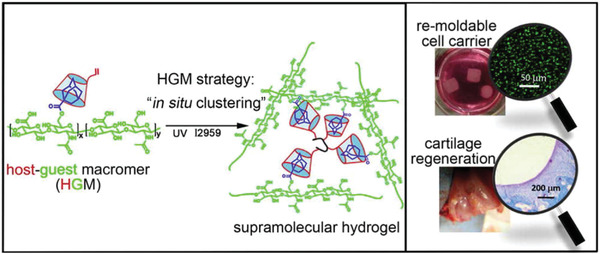
Supramolecular gels prepared applying a host–guest macromer (HGM) approach, consisting in the assembly of adamantane‐modified hyaluronic acid and monocrylated β‐CD followed by photo‐crosslinking. The gels exhibited self‐healing and ability to be remolded into freestanding 3D constructs. Reproduced with permission.^[^ ^168^ ^]^ Copyright, 2016 American Chemical Society.

CD‐based nanogels hold great promise in regenerative medicine, particularly in the controlled release of growth factors, cytokines, and morphogenetic factors, and in modifying their inner architecture for controlling cell behavior.^[^ ^157^, ^158^ ^]^ Feng et al. developed nanoemulsified CD gels loaded with kartogenin (KGN) to promote chondrogenic differentiation of stem cells, stimulating articular cartilage regeneration.^[^ ^159^ ^]^ In cardiac tissue engineering, chitosan/β‐CD nanogels loaded with adenosine were used as coating materials for synthetic blood vessels, promoting endothelization and minimizing thrombosis and hyperplasia risks.^[^ ^160^ ^]^ CD‐based nanogels have also shown potential as non‐viral gene delivery vectors to enhance tissue repair and angiogenesis.^[^ ^161^ ^]^ Additionally, they can create composites with inorganic salts for bone‐like components, potentially developing cements capable of promoting calcification and delivering growth factors.^[^ ^162^ ^]^ The host–guest supramolecular complex of CD‐based nanogels can serve as a biodegradable scaffold, with the ability to adjust drug release rates through enzyme‐degradable linkers or copolymer fortification.^[^ ^163^ ^]^ Moreover, CD‐based nanogels with zipper‐like assemblies and supramolecular poly(pseudo)rotaxane structures exhibit exceptional viscoelastic characteristics applicable to cartilage regeneration and improved viscoelasticity in intra‐articular injections (**Figure** **7**).^[^ ^164^, ^165^, ^166^, ^167^ ^]^

Polyrotaxanes (PR) are mechanically interlocked molecules composed of CDs and a linear polymer guest (poly(PEG)), forming “slide‐ring materials” where CD cross‐links freely slide on the polymer axis between adjacent CDs, extending the polymer. Jiang et al. improved this system by using hydroxypropyl‐α‐CDs (PR02) to avoid aggregations typical of native CDs and maintain network integrity under strong deformation.^[^ ^94^ ^]^ The resulting nanogels exhibited extreme stretching (1600% elongation, 1 MPa stress resistance), making them ideal for applications requiring high strength and fatigue resistance, such as synthetic cartilage and skin.^[^ ^94^ ^]^

In situ forming scaffolds are fluid materials applied to the human body with minimally invasive techniques.^[^ ^169^ ^]^ These scaffolds have various compositions by copolymerizing CDs with PEG or poly(ethylene oxide)‐poly(propylene oxide)‐(poly(ethylene oxide) (PEO‐PPO‐PEO).^[^ ^157^ ^]^ The interactions among poly(pseudo)rotaxanes are reversible, and these gels show thixotropic‐like behavior.^[^ ^170^ ^]^ Minor stress disrupts CD interactions, lowering viscosity, and facilitating easy injection through a small needle. Once stress is removed, the gel reassembles. PEO‐PPO‐PEO gels with α‐CDs exhibit mechanical characteristics dependent on temperature and the crystalline arrangement of threaded CDs.^[^ ^171^ ^]^

#### 3.2.3 Biosensing

CD‐based nanogels play a crucial role in sensing various parameters for personalized medicine, aiding in precise drug administration. These nanogels serve as carriers for hydrophobic probes used in diagnostic techniques, improving probe stability, response time, and cytocompatibility.^[^ ^172^ ^]^ For example, Wen et al. developed a colorimetric method using β‐CD‐modified AuNPs to detect dopamine.^[^ ^173^ ^]^ Our group is investigating CD‐based nanogels for dual sensing of curcumin, an important anticancer drug, with promising initial results. Recently, Sortino and our group reported the supramolecular assemblies of fluorescent nitric oxide photoreleasers with ultrasmall CD nanogels. The activatable and persistent fluorescence emissions of the NO‐photoreleasers were found to be useful for monitoring their interactions with the Gram‐positive methicillin‐resistant *Staphylococcus aureus*, whose growth was significantly inhibited by γ‐CD nanogel/NO‐photoreleaser assemblies upon light irradiation.^[^ ^174^ ^]^ Overall, the biomedical applications of nanogels are actively researched, and molecular imaging methods enhance our understanding of nanogel interactions with living matter, as discussed in the following chapters.

## 4 Radiolabeled Nanogels

Molecular imaging is a noninvasive medical imaging technique and facilitates fundamental information about functional, molecular, and structural processes insight an organism. The visualization of infectious or cancerous diseases in real time with precise accuracy can be obtained by conventional or modern medical imaging modalities such as computer tomography (CT), magnetic resonance imaging (MRI), optical imaging (OI) (e.g., fluorescence‐guided surgery, photoacoustic imaging, etc.) as well as nuclear imaging (e.g., single‐photon‐emission computed tomography (SPECT) and positron emission tomography (PET)).^[^ ^43^, ^175^, ^176^, ^177^, ^178^ ^]^ The information gained is not only beneficial for (early) diagnosis, but also to develop treatment options. In addition, these modalities are essential to understand the fate of novel imaging probes regarding their biodistribution, trafficking, and accumulation pattern in vivo. All modalities exhibit different advantages and disadvantages regarding their temporal and spatial resolution, sensitivity, and tissue penetration depth. Several reviews discuss the benefits and drawbacks of imaging modalities elsewhere.^[^ ^179^, ^180^ ^]^ The combination of these modalities, termed as multimodal imaging, leads not only to synergistic effects, but also overcomes their limitations of properties being used as single instruments. For example, the combinatorial application can result among other in higher image resolution gaining anatomical and quantificational data of cellular processes with deeper penetration depth.

Since the last decades, nanogels were decorated and encapsulated with different functionalities being used for different imaging modalities. Especially, several groups developed nanogels as contrast agents for MRI,^[^ ^181^, ^182^, ^183^, ^184^ ^]^ OI,^[^ ^112^, ^185^, ^186^, ^187^, ^188^ ^]^ or as PET/SPECT probes.^[^ ^179^ ^]^ Degradation and biomaterial–tissue interaction of gelatin‐based nanogels were investigated in vivo by multimodal preclinical imaging (MRI, OI, PET).^[^ ^189^ ^]^ In addition to that, dual‐labeled nanogels have been developed for MR/fluorescence,^[^ ^190^, ^191^, ^192^ ^]^ PET/fluorescence^[^ ^193^, ^194^ ^]^ and very recently for ultrasound/fluorescence imaging.^[^ ^112^ ^]^ In particular, ultrasound‐switchable fluorescence imaging (USF)^[^ ^195^, ^196^ ^]^ is a new hybrid modality combining near‐infrared fluorescence and ultrasound imaging to employ nanogels as unique USF imaging agents. In this review, however, we focus on radiolabeled nanogels mainly being used as nuclear imaging probes, but recently also developed for radionuclide therapy.

### 4.1 Radionuclides

Nuclear imaging is considered as a noninvasive in vivo imaging technique referring to single photon emission computed tomography (SPECT) and positron emission tomography (PET). Both techniques depend on the physical properties of a radionuclide. PET is used when a radionuclide emits positrons (β^+^), whereas SPECT is used when a radionuclide emits gamma radiations.^[^ ^48^, ^49^, ^50^, ^175^, ^178^ ^]^ The unique advantages that nuclear imaging techniques offer are quantification of the pharmacological behavior of radiolabeled agents, superior sensitivity (10^−10^–10^−12^ m) and the unlimited penetration depth in tissues in comparison to other imaging modalities. A rotating gamma camera statistically detects the events of gamma photons of a certain energy level. Collimators are used to filter the radiation except those ones coming from a certain direction. PET cameras detect two photons at a distinct energy of 511 keV in 180° (coincidence detection made up by annihilation of positrons with nearby electrons) and localizes more radiation events in time. That explains the loss of spatial resolution especially for clinical SPECT scanners (5–12 mm) in comparison to clinical PET scanners (3–6 mm).^[^ ^177^, ^178^ ^]^

Over the years, a range of SPECT and PET radionuclides have entered the clinics. The most diagnostically relevant SPECT radionuclides are technetium‐99m, indium‐111, and iodine‐123 whereas PET radionuclides such as fluorine‐18, gallium‐68, zirconium‐89 and copper‐64 have also become indispensable (**Table** **1**). Each radionuclide exhibits unique nuclear properties, which must fit the pharmacological profile of the targeting molecule. Small molecules or peptides show a rapid biodistribution pattern, blood, and renal clearance. Therefore, radionuclides with short half‐lives (e.g., ^18^F, ^68^Ga, etc.) are preferred, whereas radionuclides exhibiting longer half‐lives (e.g., ^89^Zr, ^111^In) are suitable to detect molecules with longer retention and circulation times (e.g., antibodies, etc.). In recent years, new radionuclides (e.g., ^197(m)^Hg, ^133^La, ^45^Ti, etc.) have been produced to exploit the nuclear properties and increase the global availability of cyclotron‐produced radionuclides particularly.^[^ ^197^, ^198^, ^199^, ^200^ ^]^ Nowadays, the focus of radionuclides shifted to ideally matched pairs, meaning not only being used for diagnostic, but also for therapeutic (theranostic) purposes. Therapeutic radionuclides emit alpha (e.g., ^225^Ac, ^149^ Tb, etc.) or beta (β^−^) particles (e.g., ^177^Lu, ^90^Y, ^161^ Tb, etc.) or Auger electrons (e.g., ^197^ Hg, ^67^Cu, ^99m^Tc, ^161^ Tb, etc.) to induce cytotoxic effects such as apoptosis or necrosis. The cell death is caused indirectly by the formation of radicals producing reactive oxygen species (ROS) or directly by the irreversible DNA damage. The therapeutic effect is dependent on the linear energy transfer (LET) of the radiation emitted which is in detail discussed elsewhere.^[^ ^49^ ^]^

**Table 1:** Radionuclides and their decay parameters as well as application (Note: gamma energies above 50 keV and over 10% are listed; EC = electron capture; IT = isomeric transition; CE = conversion electrons).

Radionuclide	Half life [h]	Decay mode (branching)/average energy of decay particle [keV]	Max. gamma energy [keV]	Application
^225^Ac	238	α (100%)/ α: 5935	–	α therapy
^61^Cu	3.3	β^+^ (61%)/ 523 EC (100%)	283 (13%) 656 (10%)	PET
^64^Cu	12.7	β^+^ (17%)/278 β^−^ (39%)/191 EC (62%)	–	PET β^−^ therapy
^67^Cu	61.8	β^−^ (100%)/141 Auger: 7.5 (7%)	91 (7%) 93 (16%) 185 (49%)	β^−^ therapy
^18^F	110	β^+^ (100%)/250	–	PET
^67^Ga	78.2	EC (100%)/ Auger: 7.5 (61%)	93 (39%) 185 (21%) 300 (17%)	SPECT
^68^Ga	1.13	β^+^ (89%)/ 830 EC (100%) Auger: 7.5 (5%)	–	PET
^197m^Hg	23.8	EC (9%) CE‐L: 150 (50%) CE‐K: 82 (20%)	134 (34%)	SPECT therapy
^197^Hg	64.1	EC (97%) CE‐L: 63 (60%) Auger L: 7.6 (71%)	77 (19%)	SPECT Auger therapy
^123^I	13.2	EC (100%)	159 (83%)	SPECT
^131^I	192.6	β^−^ (100%)/182 Auger: 3.4 (6%)	284 (6%9 365 (82%) 637 (7%)	β^−^ therapy
^111^In	67.2	EC (100%) Auger: 19.3 (16%)	171 (91%) 245 (94%)	SPECT
^133^La	3.9	β^+^ (7%)/460 EC (100%)	–	PET
^177^Lu	159	β^−^ (100%)/134 Auger: 6.2 (9%)	113 (6%) 208 (10%)	β^−^ therapy SPECT
^44^Sc	4.0	β^+^ (94%)/632 EC (100%)	–	PET
^47^Sc	80.4	β^−^ (100%)/162	159 (68%)	β^−^ therapy SPECT
^149^Tb	4.12	α (17%)/ α: 3967 β^+^ (7%)/ β^+^: 720 Auger L: 4.8 (64%) Auger K: 35 (5%)	165 (27%) 352 (30%) 389 (19%)	α therapy
^152^Tb	17.5	β^+^ (20%)/ 1140 EC (100%) Auger L: 4.8 (59%) Auger K: 35 (5%)	271 (10%) 344 (64%)	PET
^155^Tb	128	EC (100%) Auger L: 5 (119%)	87 (32%) 105 (25%)	SPECT
^161^Tb	165	β^−^ (100%)/154 Auger K: 5.2 (88%) Auger K: 37.2 (2%)	49 (17%) 75 (10%)	β^−^ and Auger therapy SPECT
^99m^Tc	6.0	IT (100%)	141 (89%)	SPECT
^45^Ti	3.1	β^+^ (85%)/ 439 EC (100%)	–	PET
^86^Y	14.7	β^+^ (32%)/ 660 EC (100%)	443 (17%) 703 (15%) 777 (22%) 1077 (83%) 1153 (31%) 1854 (17%) 1920 (21%)	PET
^90^Y	64	β^−^ (100%)/ 932	–	β^−^ therapy
^89^Zr	78.4	β^+^ (23%)/ 396 EC (100%)	909 (%)	PET

An ideal matched pair (e.g., ^123/124/125/131^I, ^64/67^Cu, ^149/152/155/161^ Tb, ^44/47^Sc, etc.) consists of radioisotopes (same element, but different atomic numbers) exhibiting properties suitable for diagnostic and therapeutic applications. It is however important that the diagnostic and therapeutic radioisotopes show identical pharmacological behavior in vivo when it comes to evaluating the radiotracer's biodistribution pattern or dosimetry.^[^ ^49^ ^]^ Clinically and scientifically relevant examples of radionuclides are depicted in Table 1.

**Figure 8:**
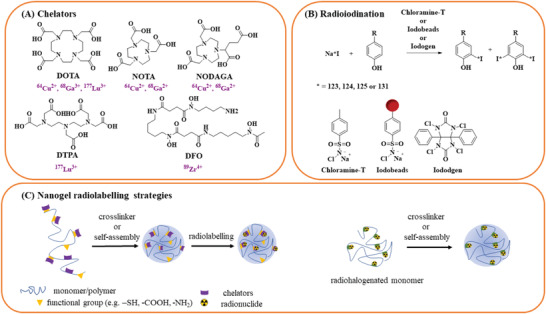
Chemical structures of various compounds used to radiolabel nanogels discussed in this review. A) Structures of metal‐based chelators (and their respective radionuclide(s) in purple) that are attached to the monomer or polymer chains. B) Common radioiodination strategy using different mediators such as chloramine‐T, iodobeads or iodogen. C) Nanogel radiolabeling strategies using chelator‐based or non‐metal radiolabeling approaches.

Nonmetallic radionuclides (e.g., ^18^F and ^123/124/125/131^I, etc.) are covalently bound to the organic compounds. These radiolabeled organic molecules are often identical or similar to the non‐radiolabeled (parent) compound and do behave pharmacologically in the same way. For example, most of the radiohalogenation of nanogels occurs with iodine radioisotopes. Widely used mediators such as chloramine‐T, iodogen or iodobeads^[^ ^201^ ^]^ have been used for the radioiodination of organic compounds carrying most likely a tyrosine residue (**Figure** **8**). Radioiodination offers favorable yields, less synthesis, and purification steps in comparison to metal‐based strategies and quick radiolabeling kinetics.^[^ ^178^ ^]^ In contrast, metal‐based radionuclides (e.g., ^99m^Tc, ^67/68^Ga, ^64/67^Cu, ^89^Zr, ^111^In, ^177^Lu, etc.) often require chelators to bind the radionuclide and consequently, these chelates alter the molecular size, weight and charge of the parent molecule (Figure 8). A high thermodynamic stability and kinetic inertness are further important aspects of metal‐based radiotracers to prevent the loss of radiometal in vivo and to avoid off‐target accumulation in healthy tissues or organs.^[^ ^177^ ^]^ For that reason, the choice of the ideal chelator for a certain metal ion is of utmost importance where several coordination aspects such as coordination number, ion radii, oxidation state, thermodynamic, and electronic properties have to be considered. Two types of chelators are available—acyclic and cyclic (special case: macrocyclic) ligands. Acyclic chelators often show a fast and efficient radiolabeling at mild temperatures, but do not meet the necessary kinetic inertness criteria to avoid the loss of radiometal in vivo. In contrast, cyclic or macrocyclic chelators exhibit a high degree of pre‐organization and rigidity. The complexes exhibit higher stabilities, but suffer from slow radiolabeling kinetics, especially at mild temperatures. Usually high temperatures (>60 °C) are needed to achieve quantitative labeling which is not suitable for heat‐sensitive nanogels or biological molecules (e.g., antibodies, DNA, etc.).

### 4.2 Radiolabeling Strategies

Many organic nanomaterials such as liposomes, dendritic polyglycerols, polymeric micelles, and viral or protein‐based nanoparticles have been radiolabeled over the past years and are reported in detail previously.^[^ ^177^, ^202^, ^203^ ^]^ There is a growing interest among researchers to study the potential of radiolabeled nanogels for nuclear applications. However, only few studies are reported so far. Whether the focus is on PET/SPECT imaging, radionuclide therapy, or tracking the payload delivery after release from nanogels, the aim is to deliver the radiotracer specifically to the target cells to obtain a low background‐to‐noise ratio and low off‐target accumulation in healthy tissues. Therefore, attempts have been made to improve the radiolabeling strategies of the radiolabeled nanogels.

In principle, the polymeric structure and the crosslinking chemistry of nanogels allows the functionalization of radionuclides via different strategies (see Section 3). Radiolabeling strategies have evolved from simple surface conjugation to incorporation of the radioactive entity.

One strategy is to modify the functional groups (e.g., amines, carboxylates, thiols, etc.) of the hydrophilic (charged) polymers with chelators before the crosslinking process takes place (Figure 8). These chelators are covalently attached to the surface via different functionalization strategies (e.g., thiol, carboxylic acid or amine conjugation) resulting in high stabilities and radiochemical yields.^[^ ^46^, ^204^ ^]^ Besides the common functionalization strategies which have been mainly reported for the functionalization of nanogels, biorthogonal click chemistry reactions (e.g., inverse‐electron‐demand Diels‐Alder reaction, strain‐promoted azide–alkyne cycloaddition) are true alternatives with respect to specific and selective radiolabeling avoiding the loss of radiometal ions in vivo.^[^ ^205^, ^206^ ^]^

On the other hand, the chelator‐based radiolabeling approach also includes the entrapment of the radiolabeled complexes into self‐assembled nanogels formed by amphiphilic polymeric networks and electrostatic repulsions.^[^ ^205^ ^]^ Which strategy is favorable depends on the structure of the chosen polymer and the existence of free functional groups suitable for bioconjugation.

### 4.3 Radiopharmaceutical Prospects

Nanosized carriers are suitable for targeting tumors via the enhanced permeability and retention (EPR) effect that localizes nanoparticles in tumors, owing to unique physiological characteristics of tumors such as abnormally high density and high permeability of blood vessels, the lack of lymphatic drainage decreasing clearance rate.^[^ ^207^ ^]^ Nanocarriers can provide additional advantageous effects for the targeting of tumors when the following features are equipped: solubilizing hydrophobic drugs, protecting the drugs from the degradation by enzymes in vivo, and controlling the rates of drug releasing.^[^ ^208^ ^]^

**Scheme 3:**
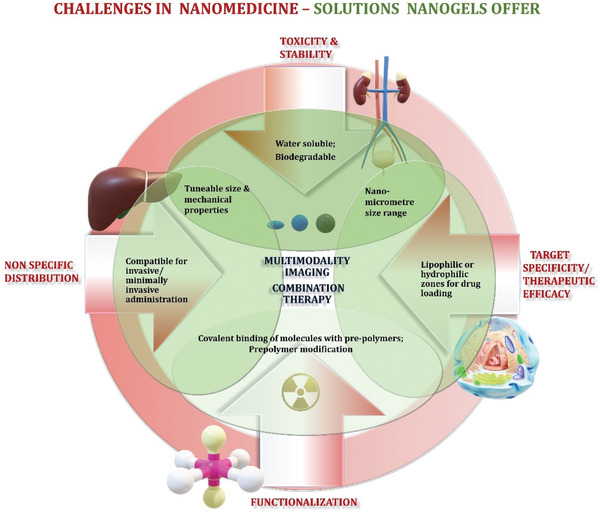
Cartoon is highlighting challenges in nanomedicine and solutions nanogels may offer to improve multimodal imaging and combinational therapy. For successful clinical translation, specific challenges in synthesis, design and delivery of nanoparticles such as functionalization of nanoparticles, toxicity, stability in vivo, nonspecific distribution, efficacy for desired application (highlighted in red text) could be addressed through the use of functionalized nanogels carrying certain properties (highlighted in green).

The in vivo efficacy of nanoparticles in disease imaging and therapy is challenged by biological barriers such as interactions with blood proteins, stability towards degradation, non‐specific uptake in reticuloendothelial organs and noncorrelated (in vitro with in vivo) interaction in disease tissue (**Scheme** **3**). To overcome these barriers, nanoparticles have been engineered with suitable intrinsic properties and functionalities. In this context, nanogels show a unique set of physical properties such as water solubility, non‐toxic polymeric composition, high quantity loading of water insoluble or soluble drugs, tunable size, and elasticity for desired pharmacokinetics and biodistribution. Not least, they mimic living tissue. The polymeric structure and crosslinking chemistry can be modulated for desired functionalization with target‐specific reporter molecules/radionuclides/fluorescent dyes or stimuli‐responsive substrates. Within this chapter, we are summarizing radiolabeled nanogels designed as PET/SPECT, dual imaging probes, or therapeutic agents. Besides, nanogels have been also developed for boron neutron cancer therapy.^[^ ^209^, ^210^, ^211^ ^]^ Until to date, there are few articles published since 2006 focusing on radiolabeled nanogels (**Table** **2**).

**Table 2:** Overview about selected properties of radiolabeled nanogels, nanogels incorporating radiolabeled guests and pre‐targeting approaches.

Nanogel matrix	Label	*D*_h_ [nm]^a)^	Zeta potential [mV]	Application	Refs.
Polyacrylamide	[^99m^Tc]Tc‐HCFU	<50	–	Drug delivery in vivo behavior	[212]
CD functionalized with camptothecin	[^64^Cu]Cu‐DOTA	40	–	In vivo behavior	[208]
Pullulan	^111^In‐ and ^18^F‐ or Alexa Fluor 467 neurotoxin BoHc/A	≈40	7 ± 0.5	drug delivery	[213]
Dextran	[^89^Zr]Zr‐DFO	13.3	–	In vivo imaging	[214]
PEGylated star polyacrylate	[^68^Ga]Ga‐NODAGA/ Alexa Fluor 488	290 ± 50	–	In vivo imaging	[215]
Polyacrylamide	^124^I‐(cyanine dye)‐labeled porphyrine	53.8	15.39 ± 0.62	In vivo behavior PDT	[193]
Chitosan	[^64^Cu]Cu‐DOTA Cyanine5.5	270	–	In vivo imaging	[194]
Polyacrylamide	[^64^Cu]Cu‐DOTA(NOTA)	63	−5.9 (−3.9)	In vivo imaging	[216]
*trans*‐Cyclooctene‐grafted cyclodextrin polyethylenimine polymer, adamantane‐grafted polyamidoamine dendrimer and adamantane‐grafted polyethylene glycol	^64^Cu‐labeled tetrazine	100	–	In vivo imaging	[217]
PEGylated star polyacrylate	[^68^Ga]Ga‐DOTA(NOTA)	200–500	−10 to −14	In vivo imaging	[218]
PEGylated star polyacrylate	[^68^Ga]Ga‐NODAGA	120	–	In vivo imaging	[219]
Carboxymethyl cellulose	[^131^I]I‐BSA	120 (BSA/CMC‐CPT) 90 BSA/CMC	≈40	Drug delivery in vivo imaging	[220]
CD NG	^99m^Tc‐labeled CD NG (targeting agent) & adamantane‐functionalized micro albumin aggregates (pre‐targeting agent)	12	–	Drug delivery in vivo imaging	[221]
5‐FU‐carbopol 934	[^99m^Tc]Tc‐5‐FU	–	–	In vivo imaging	[222]
CD NG	^111^In‐labeled CD NG (targeting agent) & ^99m^Tc‐labeled pre‐targeting agents	–	–	Drug delivery In vivo imaging	[223, 224]
Polyacrylamide	[^177^Lu]Lu‐DOTA	380		Drug delivery	[225]
PEG‐polyacrylate	[^99m^Tc]Tc‐PEO‐PAA‐folic acid	98	50	In vivo imaging	[226]
PHEG‐Tyr (polypeptide)	[^125^I]I‐PHEG‐Tyr	230		SPECT	[227]
Chitosan‐bovine serum albumin	[^99m^Tc]Tc‐CS/BSA‐(MPR)	341–535	10.3–18.9	Drug delivery	[228]
PEGylated star polyacrylate	[^125^I]ITdU	129 ± 31	−25.6	Drug delivery in vivo behavior	[229]
PEGylated star polyacrylate	[^68^Ga]Ga‐DOTA	280–310	−27 to −24	In vivo imaging	[230]
Polyethyleneimine	[^131^I]I‐PBA‐PHP	389 ± 7.1	20 – 40	SPECT radiotherapy	[231]

^a)^ *D*_h_ = hydrodynamic diameter measured by DLS in aqueous solution; n.d. = not determined.

#### 4.3.1 PET/SPECT Imaging

Radiolabeling of nanogels was first reported by Soni et al.^[^ ^212^ ^]^ The crosslinked *N,N’*‐methylenebisacrylamide nanogels (50 nm in size) were synthesized using *N*‐isopropylacrylamide (NIPAAM) and *N*‐vinylpyrrolidone (VP) polymers to encapsulate 1‐hexylcarbamoyl‐5‐fluorouracil (HCFU), an antineoplastic prodrug of 5‐fluorouracil (FU). Polysorbate 80‐coating (optimized concentration 1% w/w) was used to facilitate the delivery of the nanogels across the blood brain barrier (BBB) and block the efflux channels on cancer cells. It has been reported that 5‐fluorouracil forms complexes with ^99m^Tc, ^[^ ^232^ ^]^ thus HCFU was radiolabeled with ^99m^Tc before entrapping the radiocomplex into the nanogel. Biodistribution and SPECT imaging were performed in strain “A” mice and rabbits (SPECT images) to visualize the organ and brain uptake of ^99m^Tc‐labeled poly(NIPAAM‐VP) nanogels with or without polysorbate 80. Both coated and uncoated radiolabeled nanogels showed high uptake after 2 h in the reticuloendothelial system (RES) such as liver (≈15% vs >17% ID/g), spleen (≈8% vs ≈10% ID/g) and also lung (2.49% vs 2.11% ID g^‐1^). Not surprising, the ^99m^Tc‐HCFU complex was not stable and dissociated in vivo. The formulation strategy using 1% w/w of polysorbate 80 in comparison to uncoated nanogel increased the circulation time of ^99m^Tc‐labeled HCFU nanogel, but was not very effective in transporting the nanogel across the BBB (showing the highest accumulation after 5 min 0.52% for coated versus 0.18% ID for uncoated radiolabeled nanogel), possibly due to high RES accumulation. Nonetheless, the authors assumed that the coating with polysorbate alters the surface properties, which in turn led to a higher brain uptake in comparison to uncoated nanogels.

Chitosan‐bovine serum albumin‐based (CS‐BSA) nanogels were synthesized by self‐assembly.^[^ ^228^ ^]^ Mupirocin (MPR), an antibacterial agent was encapsulated to act as an alternative dermal drug delivery system to cure skin infections or wounds. Carbopol 940 was added to increase the adhesive capacity and gelation. The group investigated the drug release, the efficacy against *Staphylococcus aureus* and tested the cytotoxicity of different formulations with and without MPR. Cell studies in a skin cell line were performed with ^99m^Tc‐labeled (of note no chelating ligand was used) CS‐BSA nanogels with or without loaded MPR and revealed a high cellular binding, no cytotoxicity and efficient drug release. However, in vivo imaging was not reported.

Nochi et al. used cholesteryl‐group‐bearing pullulan (CHP) and cationic CHP (cCHP which contain 0.15 amino groups/glucose) polymers, which self‐assemble to nanogels in water.^[^ ^213^ ^]^ These nanogels were developed as delivery vehicles for cancer vaccine development to encapsulate the vaccine antigen BoHc/A (*Clostridium botulinum* type‐A neurotoxin subunit antigen Hc) mainly by hydrophobic interactions and then to release the active protein in the native form. The intranasal administration of BoHc/A from cCHP revealed that the antigen‐delivery efficacy is higher for the cationic nanogel. It interacted more strongly with the negatively charged cell layer membranes and therefore was taken up by endocytosis. In vivo PET imaging was performed to track the delivery of the cCHP nanogel carrying the ^18^F‐radiolabeled BoHc/A after intranasal administration and to study accumulation in the nasal mucosa and central nervous system (CNS). Biodistribution study of ^111^In‐labeled BoHc/A‐cCHP and BoHc/A demonstrated that the antigen retained longer in the nasal tissue when trapped in the cCHP than without any formulation (≈20 SUV vs ≈2 SUV after 48 h, standardized uptake value).

Gupta et al. developed 18 nm sized amine‐functionalized polyacrylamide nanogels for the delivery of an ^124^I‐labeled porphyrin derivative, known as a photosensitizer used in photodynamic therapy.^[^ ^193^ ^]^ The ^124^I‐labeled porphyrin was obtained on reacting an intermediate trimethyl tin precursor with Na^124^I. Whole body PET images in Balb/c mice bearing subcutaneous colon26 tumor revealed significant accumulation in the tumor which increased within 72 h (RUV 3.2 after 24 h and RUV 9.8 after 72 h; relative uptake value).

Majumdar et al. synthesized 13 nm sized amino‐dextran nanoparticles crosslinked using epichlorohydrin for detecting phagocytic activity in inflammatory arteriosclerotic plaques via PET imaging.^[^ ^214^ ^]^ Flow cytometry of cells isolated from excised aortas showed that the nanogels were predominantly taken up by macrophages in comparison to neutrophils and lymphocytes. The exact reason for the macrophage specificity was not clear and seemed to be correlated with the size of the nanogels. The amino groups at the surface of the dextran nanoparticles (DNP) were equipped with desferoxamine and then radiolabeled with the PET radionuclide zirconium‐89. The ^89^Zr‐labeled DNP showed phagocytic activity in inflammatory arteriosclerotic plaques via PET imaging. However, biodistribution data in C57B1/6 mice revealed high RES uptake (11.1 ± 1.4% ID g^‐1^ in liver, 10.3 ± 1.8% ID g^‐1^ in spleen) and low uptake in the aorta (1.2 ± 0.1% ID g^‐1^) or heart (1.2 ± 0.1% ID g^‐1^) after 48 h of intravenous injection. Previous studies comparing desferoxamine (DFO) and other chelators for ^89^Zr^4+^ complexation, ^[^ ^5^ ^]^ suggest that the in vivo stability of [^89^Zr]Zr‐DFO complex is affected by its low kinetic inertness which causes the free ^89^Zr^4+^ to be accumulated in bone. However, data on the bone uptake to confirm the complex stability in vivo was not reported by Majumdar et al. There are suitable chelators known for zirconium‐89 where complete saturation of the coordination sphere and spatial embedment of the central ion is achieved. Chelators such as DFO*, 3,4,3‐LI(1,2‐HOPO) and DOTA exhibit enhanced stabilities in vivo compared to the hexadentate DFO.^[^ ^233^, ^234^, ^235^, ^236^ ^]^

Singh et al. developed self‐assembled amphiphilic PEG‐based nanogels (NG) (270 ± 50 nm) crosslinked via disulfide bonds.^[^ ^215^ ^]^ The star‐shaped PEG‐based pre‐polymers carried two hydroxy and four thiol terminal groups. The thiol groups not only offer the covalent attachment of the chelating agent NODAGA (two NODAGA molecules per pre‐polymer) and the fluorescent dye Alexa Fluor 488 via Michael‐type addition reactions, but also promote the crosslinking of the nanogel formation by self‐assembly. The hydroxy groups were reserved for further functionalization to targeting molecules. Of note, the nanogels will be degenerated after cell uptake, because of the reduction of the disulfide bonds in the cytosol. Radiolabeling of the NODAGA‐labeled nanogels with the generator radionuclide gallium‐68 was straightforward achieving high radiochemical yields at room temperature (>95%). In vivo evaluation was not reported, however in vitro investigations on the internalization capacity of nanogels by phagocytosing macrophages have been demonstrated. Therefore, the phagocytosis rate was investigated by stimulated and unstimulated human leukemia monocyte cells (THP‐1) treated with Alexa Fluor 488/NODAGA nanogels. The stimulated macrophages were treated with phorbol‐12‐myristate‐13‐acetate (PMA), and showed an enhanced internalization in comparison to unstimulated THP monocytes (73% and 9%). This proof‐of‐principle study was a prerequisite to investigate the biodistribution and pharmacological behavior of radiolabeled degradable PEG‐based nanogels in vivo later by the same group.

Singh et al. investigated the role of nanogel elasticity and their corresponding influence in vivo.^[^ ^230^ ^]^ They used a similar synthesis strategy and nanogels as published in 2013.^[^ ^215^ ^]^ By tuning the number of the arm length of the thiol‐groups in the pre‐polymer, the elasticity could be varied between 30 and 95 kPa. In addition, two distinct reducible nanogels exhibiting 37 kPa (soft nanogel) and 95 kPa (hard nanogel) have been chosen for PET imaging to investigate the uptake by macrophages and their influence on the blood circulation time. The pre‐polymers were functionalized to maleimide‐DOTA enabling radiolabeling with gallium‐68. PET images and biodistribution data in Balb/c mice at 4 h post‐injection revealed that the soft reducing nanogels demonstrated slightly lower liver (≈3% ID g^‐1^ vs ≈5% ID g^‐1^) and spleen (≈1% ID g^‐1^ vs ≈2% ID g^‐1^) uptake in comparison to the radiolabeled reducible hard nanogels. Importantly, the blood circulation time of the soft reducible nanogels was enhanced indicating that the deformation of the nanogels during phagocytosis reduces the cellular uptake.^[^ ^237^ ^]^ It is worth mentioning that no difference in the biodistribution profile was observed for soft reducible and soft non‐reducible nanogels. All radiolabeled nanogels were excreted via the renal pathway, which is of uttermost importance to reduce the nephrotoxicity being used as a therapeutic agent. For that reason, the authors will investigate the suitability of these nanogels for peptide receptor radionuclide therapy (PRRT) in a future study.

**Figure 9:**
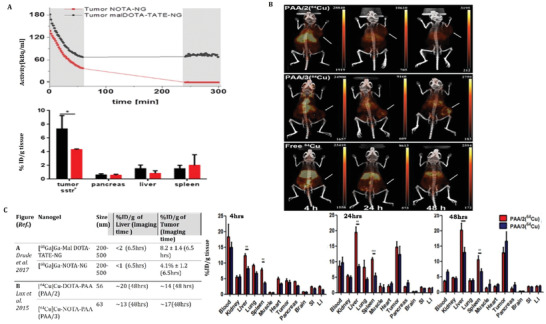
In vivo behavior of intravenously administered large and small radiolabeled nanogels in animal models: Target (cancer) and non‐target (liver, spleen) accumulation of A) µ‐PET measurements and biodistribution studies of [^68^Ga]Ga‐malDOTA‐TATE‐sPEG nanogels (black) and [^68^Ga]Ga‐NOTAsPEG nanogels (red) in Balb/c AR42J tumor‐bearing nude mice. Reproduced with permission.^[^ ^218^ ^]^ Copyright 2017, American Chemical Society. B) PET/CT images at 4, 24, and 48 h after intravenous injection of [^64^Cu]Cu‐DOTA‐PAA (2) and [^64^Cu]Cu‐NOTA‐PAA (3) in 4T1 mice bearing murine mammary carcinoma tumor (white arrow indicates the tumor). Reproduced with permission.^[^ ^216^ ^]^ Copyright 2015, Ivyspring International Publisher. C) Table highlighting the importance of targeting mechanism for above mentioned large and small nanogels which leads to an understanding that both RES accumulation and renal clearance can be achieved with large sized nanogels via active targeting of the nanogels and allowing their degradation (e.g., GSH induced in **A**) into smaller sized particles in the extracellular environment.

Lux et al. synthesized polyacrylamide‐based nanogels (PAA) crosslinked with three chelators DOTA, DTPA, and NOTA.^[^ ^216^ ^]^ These candidates were radiolabeled with copper‐64 for PET imaging. Of note, the radiolabeling procedure with copper‐64 required heating at 70 °C for 2 h, which would not be suitable for heat‐labile formulations. Instead of modifying pre‐polymers with chelators, ^[^ ^215^ ^]^ the cross‐linkers were modified with chelating ligands to bind the radionuclide of choice. Inverse emulsion polymerization of acrylamide with the corresponding cross‐linkers led to the formation of the respective nanogels (DTPA‐PAA, DOTA‐PAA, and NOTA‐PAA). The hydrodynamic diameter of all three nanogels was 65, 56, and 63 nm. The terminal groups at the surface of the nanogels stayed available for further functionalization, e.g., tumor targeting groups. In vivo experiments in Balb/c mice containing subcutaneous 4T1 murine mammary carcinoma were only performed with the [^64^Cu]Cu‐DOTA‐PAA and [^64^Cu]Cu‐NOTA‐PAA nanogels since the acyclic radiolabeled DTPA‐PAA showed poor kinetic inertness in mouse serum after one hour. PET images of both radiolabeled nanogels showed similar uptake values after 48 h, but revealed a slightly higher tumor uptake for [^64^Cu]‐DOTA‐PAA with 6.95% ID/g in comparison to [^64^Cu]Cu‐NOTA‐PAA with 4.3% ID/g (**Figure** **9**b). Biodistribution data showed an enhanced accumulation in the liver (≈20% vs ≈13% ID g^‐1^) and spleen (≈10% versus ≈8% ID/g) for both [^64^Cu]Cu‐DOTA‐PAA as well as [^64^Cu]Cu‐NOTA‐PAA after 48 hours. However, the direct comparison of the two different passively targeted ^64^Cu‐chelates revealed significantly lower RES uptake and higher accumulation of [^64^Cu]Cu‐NOTA‐PAA in the primary tumor and in the metastatic sites. Low RES uptake (5–8%) and high accumulation in metastatic tumors (≈30%) with the passive targeting approach, warrants further evaluation of ^64^Cu‐labeled NOTA nanogels for improved tumor imaging using an active targeting approach.

An active as well as passive tumor targeting approach for radiolabeled nanogels was demonstrated by Drude et al.^[^ ^218^ ^]^ The group modified star‐shaped PEG polymers bearing thiol groups, in a similar way with maleimide‐DOTA‐TATE, as reported by Singh et al.^[^ ^215^ ^]^ The somatostatin‐derived peptide (TATE) addresses somatostatin subtype 2 receptors (SstR2) overexpressed on neuroendocrine tumors enabling active targeting. These redox‐sensitive nanogels are prone to degradation mainly in the tumor environment, because of altered redox potentials leading among others to enhanced concentrations of glutathione and therefore, are the reason for passive targeting. The synthesized nanogels (malDOTA‐TATE) were radiolabeled with gallium‐68 at 95 °C and pH 5.0. [^68^Ga]Ga‐malDOTA‐TATE and ^68^Ga‐NOTA‐labeled nanogels served as controls. The biodistribution and pharmacokinetics of the nanogels were investigated in healthy Wistar rats and AR42J xenografted Balb/c nude mice (Figure 9a). Uptake by cancer cells, blood circulation as well as renal clearance was observed to be slower than for [^68^Ga]Ga‐malDOTA‐TATE resulting from the larger size of the nanogels. Elevated intracellular GSH levels in the tumor tissue caused the degradation of the nanogel forming smaller pre‐polymers (size of about 3 nm) and, thus improved the penetration to deeper cell layers. Of note, immune‐histological staining with TATE‐specific antibody confirmed a more homogeneous distribution of the [^68^Ga]Ga‐malDOTA‐TATE nanogel in comparison to [^68^Ga]Ga‐malDOTA‐TATE. Higher tumor accumulation of the [^68^Ga]Ga‐malDOTA‐TATE‐nanogels in AR42J Balb/c mice was observed 6.5 h after injection compared to non‐targeting [^68^Ga]Ga‐NOTA‐nanogels (8.2% ± 1.4% ID g^‐1^ versus 4.1% ± 1.2% ID g^‐1^). Liver and spleen accumulation were less than 2% ID g^‐1^ for both.

Drude et al. demonstrated that the initial renal clearance of redox‐sensitive nanogels as reported before was contributed by GSH triggered nanogel dissociation due to disulfide bond cleavage in the liver and in the blood. Interestingly, this was not affected by the size of the nanogels.^[^ ^219^ ^]^ Similar clearance kinetics were observed for all sizes of nanogels used in the study. The transient inhibition of GSH synthesis with a non‐toxic amount of buthioninsulfoximin (5.5 × 10^−3^ m BSO per kg body weight) injected before i.v. administration of ^68^Ga‐NODAGA nanogels led to reduction in the elimination by >40%. The authors conclude that nanogels resistant to degradation have enhanced circulation half‐lives and therefore might show different pharmacokinetics. Importantly, BSO acts as a potential radiosensitizer increasing the amount of reactive oxygen species in tumor cells, which might be beneficial for combinatorial therapeutic approaches.

Recently, the group of Ulański reported radiation‐induced (e.g., γ‐rays or electron beam) synthesis of internally crosslinked polyacrylic acid (PPA) nanogels bearing carboxylic acid groups for functionalization with DOTA‐labeled bombesin molecules.^[^ ^225^ ^]^ The bombesin peptide addresses the gastrin‐releasing peptide receptor (GRPR) overexpressed on many tumors such as breast, pancreas or prostate cancer. DOTA is able to bind to a range of trivalent metal ions such as ^68^Ga^3+^, ^89/90^Y^3+^ or ^177^Lu^3+^ (see Table 1), which fall under the theranostic radionuclide category. The DOTA‐bombesin nanogels with a molar ratio of PAA nanogels to bombesin derivative: 100/1 (≈380 nm in size) were efficiently radiolabeled with ^177^Lu^3+^ (>95% radiochemical yield) achieving the highest possible specific activity of 2.7 GBq mg^−1^. However, this proof‐of‐concept study focused only on synthesis, functionalization and radiolabeling conditions of DOTA‐bombesin labeled PAA nanogels. The biological behavior (in vivo or in vitro) was not investigated.

In the same year, Soliman et al. reported synthesis of polyethylene oxide‐polyacrylic acid (PEO‐PAA) based nanogels using γ‐radiation induced polymerization strategy.^[^ ^226^ ^]^ The PEO‐PAA nanogels were radiolabeled with technetium‐99m without any chelator and folic acid was electrostatically bound to the radiolabeled nanogels forming 98 nm [^99m^Tc]Tc‐PEO‐PAA‐folic acid particles. Effect of radiation dose, composition and concentrations of the reactants was investigated. Biodistribution profiles were studied in both healthy and tumor‐bearing (Ehrlich ascites carcinoma) mice. The data revealed a high uptake of the radiolabeled nanogels in both mice cohorts after two hours of intravenous injection. Accumulation in the tumor‐bearing mice group revealed very high accumulation in the lung (≈20% ID g^−1^), liver (≈15% ID g^−1^), intestine (≈20% ID g^−1^) and kidneys (≈40% ID g^−1^).

Another synthesis strategy was reported by Oleshchuk et al.^[^ ^227^ ^]^ The group prepared a polypeptide‐based nanogel (called as PHEG‐Tyr) crosslinked via horseradish peroxidase/hydrogen peroxide using inverse miniemulsion polymerization. This study described the usage of three different surfactants (sorbitan monooleate (SPAN 80), polyoxyethylenesorbitan trioleate (TWEEN 85), and dioctyl sulfosuccinate sodium salt (AOT)), the effect on nanogelation and the surfactant's influence on the size and morphology. Furthermore, they radiolabeled the PHEG‐Tyr nanogel with iodine‐125 using iodobeads to investigate the pharmacological behavior in vivo using SPECT. The most effective stabilization of PHEG‐Tyr nanogels was achieved in the presence of 20 wt% SPAN 80 exhibiting a spherical porous shape with a size of ≈230 nm and therefore this batch was selected for in vivo imaging. The in vivo SPECT study and blood clearance analysis revealed that the nanogels were rapidly cleared within 2 hours. After almost 24 h, no activity was detected in any tissue or organs demonstrating the complete clearance from the bloodstream and continuous excretion via renal pathway. The results indicated that the radiolabeled nanogels were degenerated to smaller particles with sizes below the renal threshold (<5 nm).^[^ ^233^ ^]^ A total dose of ≈24%–10% of the ^125^I‐labeled PHEG‐Tyr nanogel injected activity remained in the blood for up to 24 h and then gradually decreased within 48 h. The authors concluded that this biocompatible and biodegradable nanogel could be an excellent cargo system trapping inhibitors inside the porous structure.

#### 4.3.2 Hybrid Imaging

Lee et al. reported about dual‐labeled glycol chitosan‐based nanoparticles (≈270 nm) used as a PET/OI imaging probe.^[^ ^194^ ^]^ The group already developed earlier a site‐selective synthesis strategy to functionalize the glycol chitosan nanoparticles to the chelator DOTA via click chemistry.^[^ ^238^ ^]^ For that purpose, they used azide‐functionalized glycol chitosan‐5β‐cholanic acid and labeled it after self‐assembling to dibenzocyclooctyne‐functionalized DOTA (DOTA‐Lys‐PEG_4_‐DBCO) and an activatable matrix metalloproteinase (MMP)‐specific peptide tagged with the fluorophore Cy5.5 (called as AMP‐DBCO; Cy5.5‐GPLGVRGK(BHQ3)‐GG‐PEG_4_‐DBCO) and a dark quencher molecule (BHQ3). The MMP‐specific peptide is known to be overexpressed in many tumors and plays an important role in tumor progression and metastasis.^[^ ^239^, ^240^ ^]^ The radiolabeling with copper‐64 was carried out before the conjugation of [^64^Cu]Cu‐DOTA‐Lys‐PEG_4_‐DBCO and AMP‐DBCO to the azide‐functionalized nanoparticles via strain‐promoted alkyne–azide cycloaddition. Whole body PET/OI images in A549 tumor‐bearing mice revealed that tumor accumulation of [^64^Cu]Cu‐DOTA‐AMP‐CNP nanoparticles gradually increased within time, plateauing at 24 h (6.2% ID g^−1^) whereas the fluorescence signal was detected as early as 1 h post‐injection, plateauing at 6 h. High accumulation in the liver and kidneys was observed at all time points, but was significantly reduced at 48 h. Biodistribution data obtained at 48 h after intravenous injection showed similar results compared to the images with highest accumulation in liver (≈15% ID g^−1^), spleen (≈10% ID g^−1^) and kidneys (≈15% ID g^−1^). Importantly, ≈9% ID g^−1^ were still found in the blood. This is the first study, which reported about nanogels as multimodal PET/OI imaging probe using the biorthogonal click reaction approach.

#### 4.3.3 Radionuclide Therapy

The group of Morgenroth evaluated the therapeutic potential of an Auger‐electron emitting ^125^I‐labeled 5‐iodo‐4′‐thio‐2′‐deoxyuridine ([^125^I]ITdU) thymidine analog against multiple myeloma.^[^ ^241^ ^]^ Auger‐electrons are highly radiotoxic when they are incorporated into the DNA.^[^ ^242^ ^]^ To overcome the limitations crossing the blood brain barrier, the same group used nanogels as delivery systems ten years later.^[^ ^229^ ^]^ In detail, poly(ethylene oxide‐*co*‐propylene oxide) pre‐polymers with terminal acrylate groups were attached to [^125^I]ITdU‐MMP2/9 conjugates forming the nanogels via miniemulsion. The MMP substrates induced nanogel enzymatic degradation and, thus subsequent release of [^125^I]ITdU into the cells. Besides, MMP‐2 and MMP‐9 are the most abundant members detected in the glioblastoma cell lines U‐87 and HT12346. To promote BBB transcytosis and intracellular delivery to glioblastoma tumor cells, the nanogels were also postfunctionalized with a cross‐reactive material 197 (CRM‐197), which is a clinically approved ligand addressing the diphtheria toxin receptor (DTR). DTR is a transport receptor in the BBB that is also expressed in glioblastoma cells. In vitro investigations on BBB models consisting of, e.g., human glioblastoma cell lines confirmed successful transcytosis across the BBB, endocytosis of the [^125^I]ITdU nanogels and efficient release of [^125^I]ITdU into glioblastoma cells showing a high rate of DNA incorporation. This new strategy might open new treatment concepts to cure glioblastoma. However, in vivo investigations have not been performed.

Nanogels’ use in cancer therapy was first reported by Liu et al.^[^ ^220^ ^]^ The hybrid nanogel platform used within this study was developed to provide a safe and effective combined chemoradionuclide therapy using ^131^I‐labeling by chloramine‐T method and camptothecin (CPT). The nanogels were synthesized by self‐assembly of carboxymethyl cellulose (CMC), ^131^I‐labeled bovine serum albumin (BSA), and loaded with CPT. The nanogels were prepared for pH triggered release of iodine‐131 into tumor cells which still showed sustained (63% at 72 hours) release of ^131^I‐labeled BSA at pH 5.0. The in vivo biodistribution pattern of ^131^I‐BSA/CMC nanogels loaded with CPT (^131^I‐BSA/CMC‐CPT) was investigated in LLC tumor‐bearing mice. Due to the EPR effect, the blood circulation for both nanogels was prolonged showing ≈15% ID g^−1^ at 24 h after injection. Tumor uptake of ^131^I‐BSA/CMC‐CPT (7% ID g^−1^) at 24 h was higher than free ^131^I (2% ID g^−1^) and retention was observed up to 72 h while elimination continued from non‐target organs (at 24 h: liver: ≈4% ID g^−1^; spleen: ≈2% ID g^−1^; kidney: ≈3% ID g^−1^). The toxicity profile of ^131^I‐labeled nanogels showed <5% hemolysis with a normal biochemical profile for 50 d after intravenously administration. The therapeutic efficacy of the ^131^I‐labeled nanogels was also studied in C57BL/6 mice bearing LLC tumors. ^131^I‐BSA/CMC‐CPT nanogels showed an improved therapeutic effect with extended survival rates and tumor growth inhibition in comparison to ^131^I‐BSA/CMC nanogels and the nonradiolabeled BSA/CMC‐CPT. The authors efficiently showed a synergistic effect when combining chemotherapy with radionuclide therapy.

**Figure 10:**
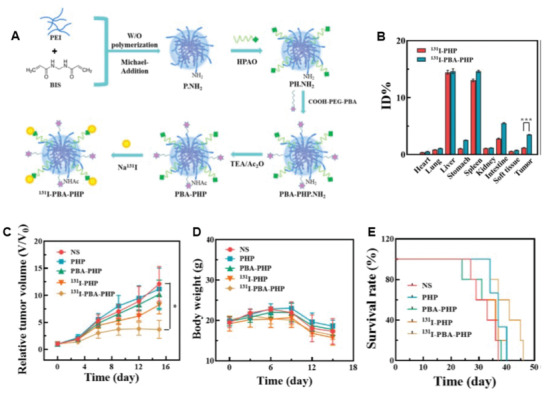
A) Synthesis scheme of [^131^I]I‐PBA‐PHP nanogels. B) Biodistribution data (% ID) of [^131^I]‐PHP and [^131^I]I‐PBA‐PHP nanogels at 16 hours post‐injection in 4T1 tumor‐bearing mice. Tumor inhibition efficacy with various treatments (NS = normal saline; PHP = non‐radiolabeled nanogels; PBA‐PHP = non‐radiolabeled phenylboronic acid nanogels; ^131^I‐PHP = radiolabeled nanogels; ^131^I‐PBA‐PHP = radiolabeled phenylboronic acid nanogels) showing C) relative tumor volume, D) body weight and E) survival rate of 4T1 tumor‐bearing mice within 47 d. Reproduced with permission.^[^ ^231^ ^]^ Copyright 2022, Frontiers.

Recently, a theranostic nanogel platform has been developed by Kong et al.^[^ ^231^ ^]^ They synthesized polyethyleneimine (PEI)‐based nanogels crosslinked with 3‐(4′hydroxyphenyl)propionic acid N‐hydroxysuccinimide (HPAO). Additionally, the PEI‐based nanogels were equipped with phenylboronic acid (PBA) ensuring active targeting to sialylated epitopes which are overexpressed on the surface of many tumors, e.g., breast cancer cells (**Figure** **10**). The iodine‐131 radionuclide was introduced to the acetylated terminal groups. SPECT imaging of ^131^I‐labeled‐PBA‐PHP nanogels was performed in Balb/c nude mice bearing 4T1 breast adenocarcinoma. The size and Zeta potential of PBA‐PHP nanogels were determined to be ≈390 nm and ≈20 mV, respectively. The nanoparticles are positively charged due to the remaining amino groups on the surface. The data revealed a tumor uptake of ^131^I‐labeled‐PBA‐PHP nanogels, which peaked at 8 h postinjection and gradually declined within 16 h. Ex vivo analysis clearly showed a significant higher tumor accumulation of ^131^I‐labeled‐PBA‐PHP compared to the non‐targeting ^131^I‐labeled‐PHP nanogels. The radiotherapeutic effect was also investigated in tumor‐bearing mice (Figure 10). Notably, tumor growth inhibition and prolonged survival were observed for ^131^I‐labeled‐PBA‐PHP nanogels in comparison to the radiolabeled control (^131^I‐PHP). In summary, the authors developed a promising approach to set up a theranostic nanoplatform.

#### 4.3.4 Radiolabeled Cyclodextrin Nanogels

In recent years, CD‐based nanogels have also gained importance in the field of nuclear imaging.^[^ ^243^, ^244^, ^245^ ^]^ As mentioned in the earlier chapters, CD nanogels have distinctive features derived from their host–guest complexation properties. This unique behavior of CDs can offer several utilities such as constructing radiolabeled drug delivery/releasing systems,^[^ ^208^ ^]^ and designing pre‐targeting platforms.^[^ ^217^, ^246^, ^247^ ^]^ In this section, we introduce several (very few) radiolabeled cyclodextrin nanogel systems published recently. Although a number of unimolecular^[^ ^248^ ^]^ and amphiphilic^[^ ^249^, ^250^ ^]^ radiolabeled cyclodextrin systems were also reported, they are beyond the scope of this review and not discussed here.

CD‐based nanogels typically do not penetrate the cell membrane but are excreted via feces.^[^ ^157^ ^]^ However, they can enter cells when functionalized with specific biological entities,^[^ ^168^ ^]^ enhancing endocytosis‐based absorption or direct cellular entrance. Functionalization also prolongs circulation in the bloodstream and may reduce immune response.^[^ ^251^ ^]^ Various functional groups, such as peptides,^[^ ^252^ ^]^ cell‐adhesive ligands,^[^ ^253^ ^]^ and aptamers^[^ ^254^ ^]^ can be introduced to such nanogels. For instance, Lu et al. developed ethylenediamine‐grafted γ‐CDs cross‐linked with gelatine, highly loaded with betulinic acid (BA) through CD cavity inclusion.^[^ ^255^ ^]^ These nanogels are capable of recognizing tumor cells via the RGD sequence grafted on gelatine, leading to tumor inhibition.^[^ ^255^ ^]^ Transferrin (TfR) receptors overexpressed in certain tumors can also be targeted using human transferrin (Tf) to enhance nanogel internalization by tumor cells.^[^ ^143^ ^]^

As is clear from the earlier chapters, CD nanogels are a suitable platform for tumor targeting based on the EPR effect. Exploiting that, Schluep et al. developed radiolabeled CD nanogels bearing the cytotoxic molecule camptothecin.^[^ ^208^ ^]^ In this structure, CD nanogels were covalently functionalized with both the antitumor agent and DOTA as chelator for ^64^Cu, resulting in an approximate diameter of 40 nm. The biodistribution of these ^64^Cu‐labeled nanogels in mice was visualized by PET imaging. The radiolabeled nanogels circulated in plasma with a biological half‐life of 13.3 h. The imaging study demonstrated that, 24 h after the injection, the nanogel was selectively accumulated in the tumor.

**Figure 11:**
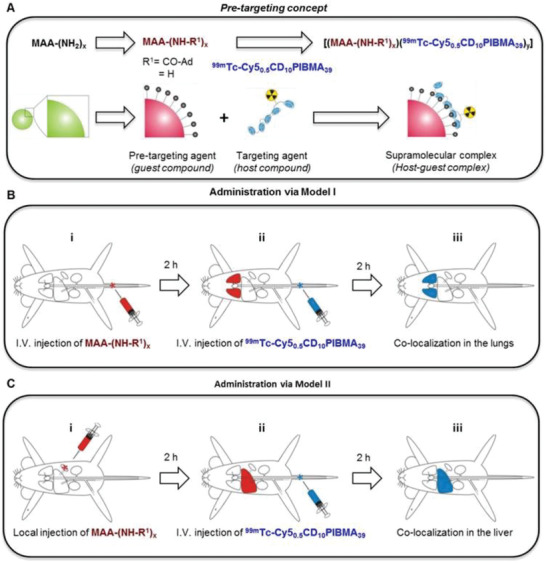
Schematic illustration of dual‐labeled (Cy5 and ^99m^Tc) PIBMA (poly(isobutylene‐*alt*‐maleic‐anhydride) nanogels containing CD units as a complementary system to recognize adamantane‐functionalized micro albumin aggregates (MAA‐Ad). Reproduced with permission.^[^ ^221^ ^]^ Copyright 2018, Ivyspring International Publisher.

Leeuwen's group applies the pre‐targeting strategy using radiolabeled CD‐based nanogels. Here, a supramolecular approach of interaction of cydclodextrin units with adamantane is exploited to achieve targeted liver radioembolization. Adamantan‐functionalized micro albumin aggregates (MAA‐Ad) as targeting component are injected both intravenously and locally into the liver. During the i.v. injection, the MAA‐Ad microspheres are enriched exclusively in the lungs. Subsequently, a dual‐labeled (^99m^Tc and cyanine dye Cy5) nanogel (*d*  = 12 nm), carrying CD units complementary to adamantane, is injected as a complementary effector agent (**Figure** **11**). This allows the accumulation, stability, and pharmacokinetics to be studied using SPECT and fluorescence imaging. The same group has also investigated the application of differently radiolabeled compounds, namely the use of ^99m^Tc‐labeled microspheres and ^111^In‐labeled CD‐containing nanogels.^[^ ^224^ ^]^ This approach has also been applied to studying the distribution of bacteria in mice.^[^ ^223^ ^]^ In this way, bacteria were functionalized with adamantane and labeled with ^99m^Tc. Subsequently, the ^111^In‐labeled nanogel was injected to interact with bacteria accumulated in muscles and liver. The pre‐targeting approach, based on the selective interaction of adamantane and cyclodextrin, in combination with differently labeled reaction partners allows the detailed investigation of the pharmacokinetic properties and offers the possibility of targeted enrichment in desired organs and tissues.

Hou et al. reported a pre‐targeting PET imaging system using key‐lock assembled supramolecular CD nanogels. These nanogels are composed of *trans*‐cyclooctene‐grafted cyclodextrin/polyethylenimine polymers (TCO/CD‐PEI), adamantane‐grafted polyamidoamine dendrimer (PAMAM‐Ad), and adamantane‐grafted polyethylene glycol (PEG‐Ad), resulting in supramolecular assembles with a hydrodynamic size of 100 ± 20 nm. These assembles were injected into mice intravenously enriched in the tumor by exploiting the EPR effect. Subsequently, a small tetrazine‐containing effector molecule, containing the chelator DOTA for ^64^Cu‐labeling was injected. This allows selective and effective bioorthogonal ligation with the TCO units of the assemblies. In this way, a high‐contrast PET image of the tumor was obtained.^[^ ^217^ ^]^

## 5 Summary and Conclusion

In particular, the last two decades have witnessed a rapid development of nanogels for various applications, but especially in the field of biomedicine. This is mainly done using a range of synthetic polymers or copolymers, some of which, such as poly(ethylene oxide), poly(glutamic acid) and poly(lactic‐*co*‐glycolic acid) are now approved for clinical use. However, natural polymers are also becoming increasingly important, for example, chitosan, gelatine, pullulan, cellulose, and dextran as a matrix for the fabrication of nanogels. The synthesis procedures have become more and more refined over the years and sophisticated synthesis protocols allow the targeted adjustment of properties such as size, charge, and shape as well as the attachment of various units such as dyes, solubilizing groups, and biological vector molecules to the surface or inside of nanogels.

More recently, CD‐based nanogels have gained importance due to their unique properties. This applies in particular to their exceptional host–guest properties, which predestines them as novel drug delivery systems for pharmaceuticals, as matrix materials for tissue engineering and new sensor systems. So far, the established fabrication methods for CD‐based nanogels are cross‐linking (covalent and supramolecular linkage) reactions, radical polymerization, and “key‐lock assembly.” This allows the properties to be adjusted over a wide range. However, these areas are still in their infancy.

Overall, nanogels open up a multitude of different application possibilities. In vivo applications in particular require the use of FDA‐approved materials as well as controlled and reliable manufacturing methods that lead to defined, uniform and highly monodisperse products and, last but not least, precise knowledge of the pharmacokinetic and pharmacodynamic properties of the nanogels. It is crucial to understand comprehensively their physiological fate particularly with respect to their absorption, distribution, metabolism, and excretion (ADME) characteristics. In this context, modern molecular imaging methods have been increasingly used in recent years. This mainly concerns established methods such as magnetic resonance and fluorescence imaging. Nuclear techniques such as SPECT and PET allow precise and reliable determination of biodistribution properties with an extremely high sensitivity. For these reasons, these methods are increasingly used. Both radiolabeled nanogels themselves and radiolabeled guest molecules such as drugs can be investigated. With regard to the latter, a very precise determination of drug release protocols in vivo is made possible. This is an enormous advantage, especially for the use of stimuli‐responsive nanogels in the study of the controlled release of guest molecules, for example when using ultrasound or in the case of changes in temperature, pH value, etc.

Overall, nuclear methods are clearly superior to other molecular imaging techniques when it comes to determining quantitative data in vivo. However, this requires the use of appropriate radiolabeling strategies of nanogels under mild conditions. In recent years, the development of suitable bifunctional chelating agents as well as new coupling strategies based on bioorthogonal chemistry has experienced a rapid development. In addition, efficient production methods for a number of unconventional and theranostic radionuclides enable the application of sophisticated imaging methods, but even the use in personalized radionuclide therapy.^[^ ^256^, ^257^, ^258^ ^]^ There is considerable development potential for radiolabeled nanogels in both areas. The radiolabeling methods of nanogels currently described in the literature with only a handful of radionuclides and, in particular, the assessment of the radiostability give broad scope for optimization. This also applies to the use of dual‐labeled nanogels. Nanogels equipped with a combination of nuclear and optical probes allow the study of in vivo processes up to the cellular level and further a clinical use in the detection of diseased tissue up to fluorescence‐guided surgery. These areas have significant clinical potential.^[^ ^50^, ^259^ ^]^ However, the use of nanogels is virtually unstudied here.

The biggest challenge for in vivo use of nanogels is biological targeting. Due to their size and surface charge, nanogels are often recognized as foreign bodies by the body's own immune system and eliminated by its phagocytic cells. A special role is played by the opsonization of the particles, i.e., the surface is coated with special proteins (protein corona) in order to be more easily recognized and taken up by phagocytic cells. Although the problem of protein corona formation has been known for 20 years now, this phenomenon is almost not considered, if at all, in connection with the development of nanogels.^[^ ^260^, ^261^, ^262^ ^]^ In order to circumvent the problem of trapping by the mononuclear phagocyte system, PEGylation of nanogels is still primarily used, despite severe side effects that are now known, such as hypersensitivity and immunogenicity.^[^ ^263^, ^264^, ^265^, ^266^, ^267^ ^]^ Here, the development of new innovative strategies is needed to make nanogels invisible to the immune system. Although not yet fully developed for clinical use, alternative methods are being used with other polymers^[^ ^268^ ^]^ such as poly(vinylpyrrolidone)^[^ ^269^ ^]^ and poly(2‐oxaline)s^[^ ^270^ ^]^ as well as polysaccharides.^[^ ^271^ ^]^ In this context, the fabrication of nearly uncharged zwitterionic surfaces is becoming increasingly important.^[^ ^64^ ^]^ Various strategies are used, such as attaching small zwitterionic molecules to the surface of nanoparticles or coating them with amphiphilic polymers.^[^ ^272^, ^273^, ^274^ ^]^ Furthermore, nanogels in the ultrasmall range, i.e., with sizes smaller than 6 nm, are becoming increasingly important. These tiny particles can rapidly circulate in the bloodstream and, equipped with suitable surface functionalization, they can reach a biological target very quickly.^[^ ^275^, ^276^ ^]^ However, this area is currently almost unexplored for nanogels.^[^ ^122^, ^125^ ^]^

The results so far in the field of nanogels for biomedical use are very encouraging. For the time being, this concerns in particular an in vitro application. Through the use of molecular imaging methods, significant progress in the understanding of in vivo behavior is expected in the following years. Nuclear molecular imaging methods are playing an increasingly important role in this. Intensive multidisciplinary cooperation between synthetic and biochemically oriented groups with radiochemists and radiopharmacists is hereby essential.

## Conflict of Interest

The authors declare no conflict of interest.
